# Specific Ion Chemistry at the Air–Water Interface of Nitrite/Nitrate-Containing Droplets

**DOI:** 10.1021/acs.est.5c15074

**Published:** 2026-03-20

**Authors:** Yoan Carreira Mendes Da Silva, Maria Angelaki, Adrien Gandolfo, D. James Donaldson, Christian George

**Affiliations:** † Universite Claude Bernard Lyon 1, CNRS, IRCELYON, UMR 5256, Villeurbanne F-69100, France; ‡ Department of Chemistry, 7938 University of Toronto, 80 George Street, Toronto, Ontario M5S 3H6, Canada; § Department of Physical and Environmental Sciences, University of Toronto Scarborough, 1265 Military Trail, Toronto, Ontario M1C 1A4, Canada

**Keywords:** air−water interface, H_2_O_2_ formation, NO_*x*_ production, HONO production

## Abstract

An increasing number of studies report on the spontaneous production of OH radicals and H_2_O_2_ at the air/water interface of aqueous droplets. However, none of these studies have investigated OH production in droplets containing nitrate (NO_3_^–^) or nitrite (NO_2_^–^) anions. Those two ions, in particular NO_3_^–^, play a key role in atmospheric chemistry. The goal of this work was to study the spontaneous production of H_2_O_2_ in water droplets containing these two anions under dark conditions. Compared to ions that we have already studied (i.e., Cl^–^ and Br^–^), H_2_O_2_ production in droplets containing NO_2_^–^ or NO_3_^–^ can be either enhanced or inhibited depending on the NO_2_^–^ or NO_3_^–^ concentrations. These findings suggest that, below a certain concentration, NO_2_^–^ and NO_3_^–^ enhance the H_2_O_2_ production due to their ability to disrupt the water structure at the interface, where at larger concentrations, a specific interfacial NO_2_^–^ or NO_3_^–^ chemistry starts to be important, producing gas-phase species such as HONO and NO_2_. By monitoring H_2_O_2_ and several other gas-phase products, we propose a mechanism to explain this specific interfacial chemistry of NO_2_^–^ and NO_3_^–^. This chemistry may not be significant during day time due the photolysis of these two anions, but it can have an important impact during night time.

## Graphical abstract

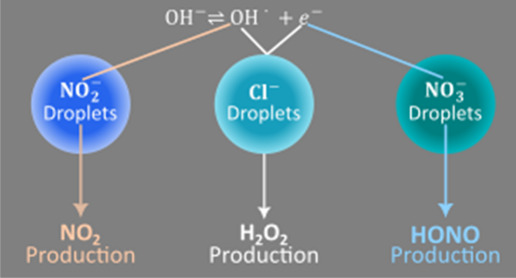

## 1 Introduction

Recently, studies have shown that “spontaneous” chemistry at the air–water interface of aqueous droplets occurs to produce OH radicals and H_2_O_2_.^1,2^ The proposed mechanism to explain this chemistry is that, at the air–water interface, OH^–^ may exist as a radical–electron pair that initiates specific chemistry (e.g., reaction [Disp-formula eqR1] below). The explanation of this dissociation is currently believed to be related to the solvation properties of OH^–^ at the interface^3,4^ or the presence of a strong electric field at the interface.^5^ Interfacial H_2_O_2_ is either produced via recombination of two OH radicals (reaction [Disp-formula eqR2]),^4,6^ or initiated by reaction between oxygen and solvated electrons (reactions [Disp-formula eqR3]–[Disp-formula eqR5]). Previous investigations have reported that, without oxygen, no H_2_O_2_ is produced at the interface of salt-containing aqueous droplets.^2,7^ This highlights the fact that the pathway involving oxygen (reactions [Disp-formula eqR3]–[Disp-formula eqR7]) is key and emphasizes the need for an electron scavenger to enable this chemistry at the air–water interface.

R1 OH−⇌OH•+e−

R2 OH•+OH•→H2O2

R3 $$O_{2}+e^{-}\to {O_{2}}^{-}$$

R4 $${O_{2}}^{-}+H^{+}⇌{{\mathrm{HO}}_{2}}^{•}$$

R5 $${{\mathrm{HO}}_{2}}^{•}+{{\mathrm{HO}}_{2}}^{•}\to H_{2}O_{2}+O_{2}$$

R6 $$H^{+}+e^{-}\to H^{•}$$

R7 $${{\mathrm{HO}}_{2}}^{•}+H^{•}\to H_{2}O_{2}$$

In this mechanism, ions are not expected to affect the H_2_O_2_ production. In our previous study, however, we reported that H_2_O_2_ production changes with the ionic composition of the droplets and follows the Hofmeister series.^8^ When ions are present at the interface, they disrupt the water structure, changing the interfacial solvation properties and promoting reactions otherwise unfavorable in the bulk, such as reaction [Disp-formula eqR1]. This suggests that the interfacial chemistry that we observed is driven by the local solvation environment. However, there has been no investigation of droplets containing either nitrite (NO_2_^–^) or nitrate (NO_3_^–^) anions. These two anions play central roles in soil biogeochemistry and atmospheric chemistry. In agriculture, NO_3_^–^ anions are the key nitrogen source for crops and are widely used in agricultural fertilizers to boost yields.^9,10^ It leads (with NO_2_^–^ to a lesser extent) to significant environmental challenges due to the ion mobility in soil and water.^11,12^ In the atmosphere, NO_2_^–^ leads to nitrous acid (HONO) formation.^13,14^ During day time, HONO photodissociates to produce OH radicals and nitric oxide (NO),^15,16^ giving rise to a major OH source in the gas phase.^17,18^ NO_3_^–^ photolysis in aqueous solution is a key process in atmospheric chemistry, producing NO_2_^–^ and nitrogen dioxide (NO_2_).^19^ This photolysis is a substantial source of nitrogen oxides (NO_*x*_), HONO, and OH radicals as well. Recent studies have reported that NO_3_^–^ photolysis is enhanced in particles.^20,21^ At night time, NO_3_^–^ is stable; however, a spontaneous dark interfacial process in NO_3_^–^/NO_2_^–^ droplets could be a potential source of HONO, NO_*x*_, and OH radicals. That is the motivation for the present investigation. For this purpose, solutions containing nitrite or nitrate salts were nebulized in a temperature-controlled reactor under dark conditions. Those droplets then traveled along the axis of a vertically aligned aerosol flow tube prior to collection and analysis of both aqueous and gaseous phases.

## 2 Materials and Methods

### 2.1 Experimental Section

Figure 1 shows a simplified schematic of the experimental setup used for the experiments of this work, which is similar to what we reported in our previous study.^7,8^

**Figure 1:**
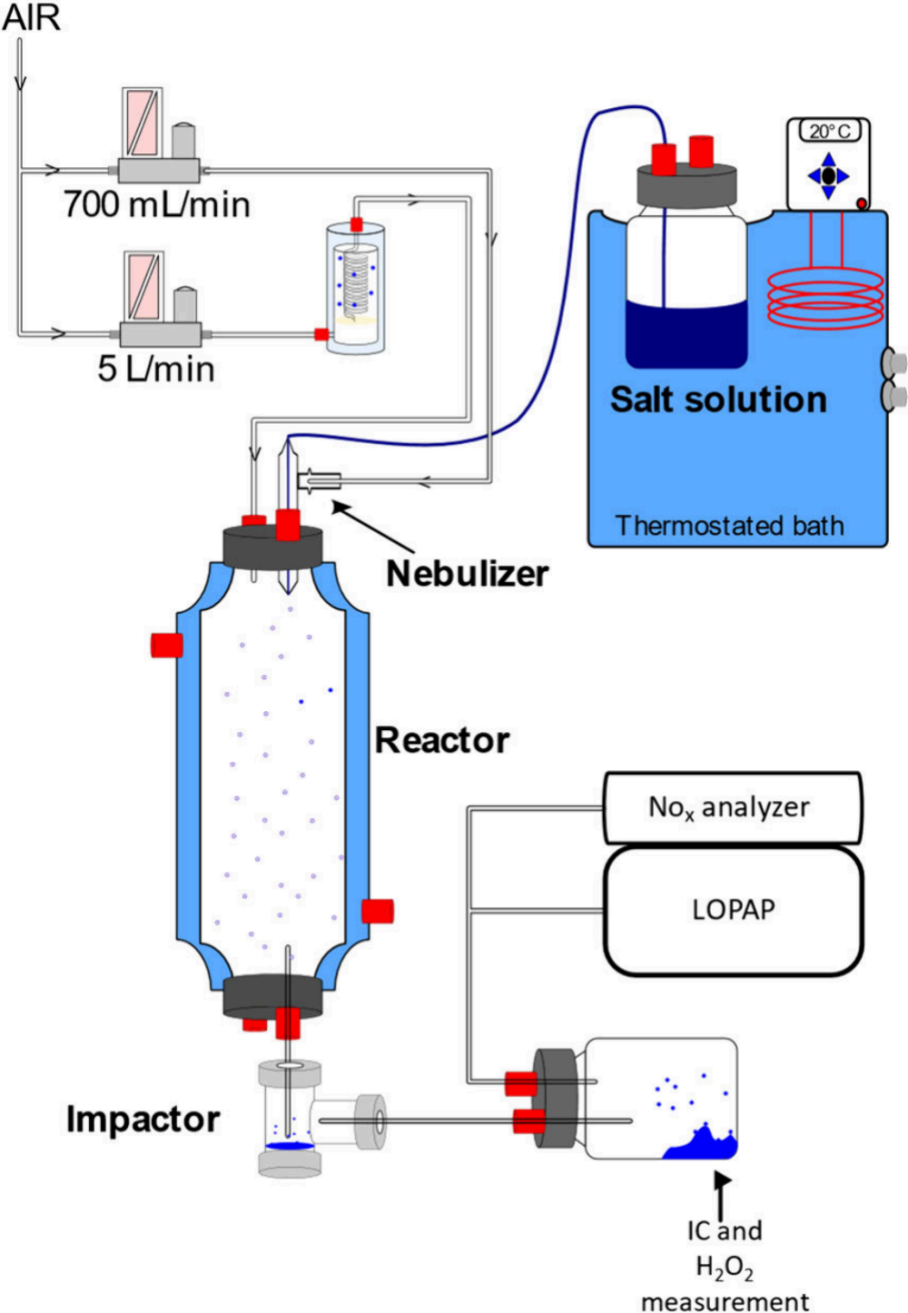
Schematic of the experimental setup.

Aqueous solutions containing sodium nitrite (NaNO_2_) or nitrate (NaNO_3_) salts were used to generate droplets into a homemade 2 L thermostated double-wall glass reactor [inner diameter (id) = 60 mm and length = 800 mm] covered with black fabric. In this reactor, a flow of droplets was injected with an additional humidified air flow (RH = 88%) using a bubbler. At the end of the reactor, a homemade impactor was connected to gather liquid droplets for liquid analysis. The residence time of the droplets inside the reactor is approximately 20 s. The pH of the solutions was 6.2 ± 0.5, and if needed, NaOH was added to increase the pH to 10.5 ± 0.2. During an experiment, the droplets were continuously generated from a Meinhard glass nebulizer. To avoid cross-contamination between the nitrite and nitrate experiments, two distinct nebulizers and dedicated glassware were used. The reactor and impactor were thoroughly cleaned before and after each experiment. In this study, the temperature was set at 290 K with a spraying time of 20 min, and an NO_*x*_ analyzer and a LOPAP are connected after the impactor to measure NO, NO_2_ (using a NO_*x*_ analyzer, CLD 88 p with PLC 860 from ECO PHYSICS), and HONO using a long path absorption photometer (LOPAP, LOPAP-03 from QUMA) in the gas phase. A particle filter protects the instruments from droplets, so that only the gas phase is measured. At the end of each experiment, a volume of 2 mL of droplets was collected in the impactor to perform H_2_O_2_ measurement and ion chromatography (IC). The H_2_O_2_ concentration was measured using a H_2_O_2_ analyzer (AL 2021 from AERO LASER). The pH of the bulk solution was measured before all the experiments with a Metrohm pH meter (model 913). Droplet size distributions were measured in selected experiments using an optical particle counter (Promo LED 2300, Palas) connected in place of the gas-phase analyzer (Figure S1). The droplets are polydisperse, with two main diameters at 240 and 440 nm, regardless of the salt solution concentration.

### 2.2 Chemicals

All chemicals were used for the experiments as received without any further purification: sodium nitrite (ReagentPlus, ≥99.0%, Sigma-Aldrich), sodium nitrate (ReagentPlus, ≥99.0%, Sigma-Aldrich), amonium nitrate (ReagentPlus, ≥99.0%, Sigma-Aldrich), sodium chloride (ReagentPlus, ≥99.0%, Sigma-Aldrich), and sodium hydroxide solution (1 mol/L, Reag. Ph. Eur., Sigma-Aldrich).

## 3 Results and Discussion

### 3.1 H_2_O_2_ Production in Droplets Containing NO_2_^–^ or NO_3_^–^

Figure 2 shows the H_2_O_2_ concentration as a function of the NO_2_^–^ or NO_3_^–^ concentration, with the results also given in Table S1. It is clear that the concentration of H_2_O_2_ decreases as the concentration of NO_2_^–^ or NO_3_^–^ inside the droplets increases. This phenomenon seems to be more pronounced in the case of NO_2_^–^, but experiments with more than 1 mM of nitrite could not be performed due to interferences between NO_2_^–^ and the H_2_O_2_ analyzer (Figure S2). This trend is totally different from that seen for the other anions that we previously studied.^7,8^ For the other anions, the H_2_O_2_ concentration increased with an increasing salt concentration until a plateau was reached. This trend was described to be similar to the Langmuir–Hinshelwood or Gibbs formalism for adsorption and reaction,^22^ possibly indicating a surface-reactive process. This is in stark contrast to what can be seen in Figure 2, which is highly suggestive of a specific chemistry occurring in the presence of NO_2_^–^ or NO_3_^–^ that inhibits interfacial H_2_O_2_ production. The H_2_O_2_ production as a function of the NO_2_^–^ or NO_3_^–^ activities is shown in Figure S3. The occurrence of reactions [Disp-formula eqR8] and [Disp-formula eqR9] discussed below could be a reason for the trend observed for NO_2_^–^ or NO_3_^–^ droplets.

**Figure 2:**
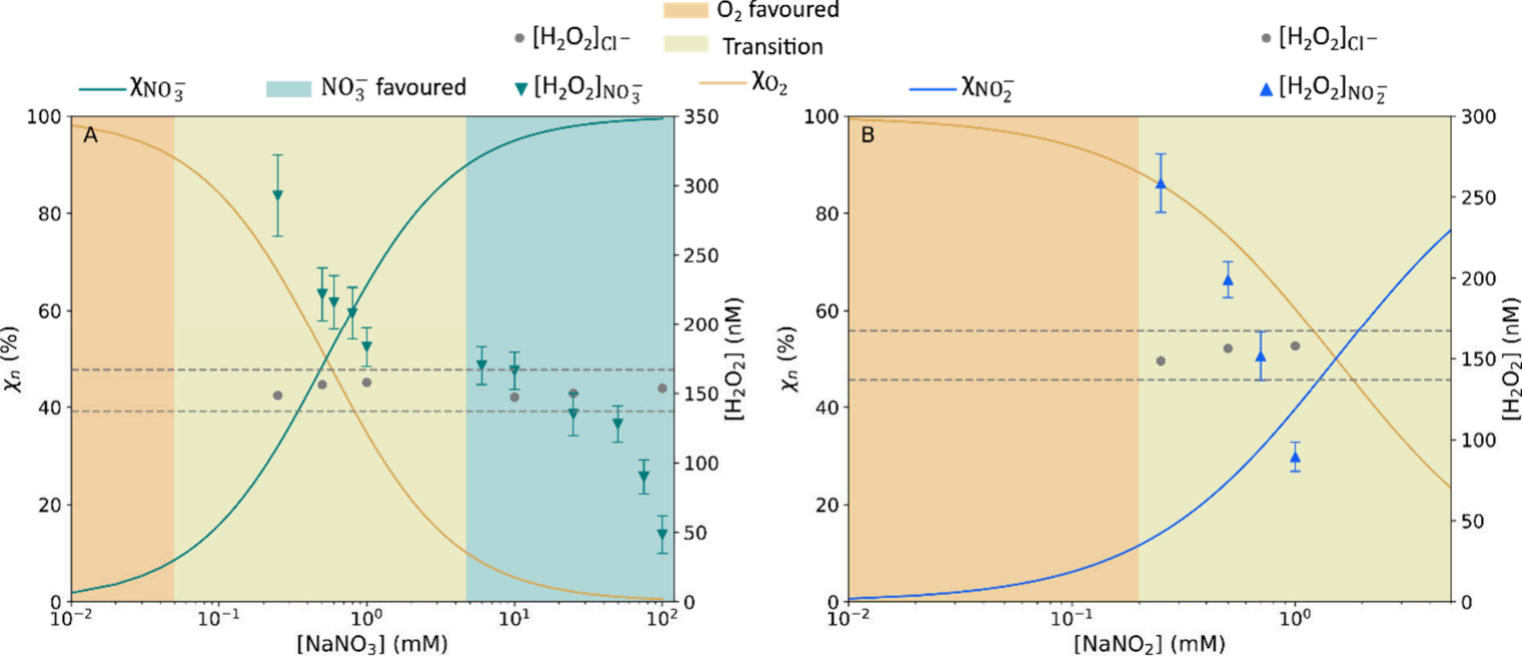
(A) Mean H_2_O_2_ concentration measured in droplets as a function of the NaNO_3_ concentration with the branching ratio (χ) of O_2_ (in orange) and NO_3_^–^ (in dark green). (B) Mean H_2_O_2_ concentration measured in droplets as a function of the NaNO_2_ concentration with the branching ratio (χ) of O_2_ and NO_2_^–^ (in blue). The colored shaded regions represent the regime of the branching ratio (O_2_ scavenging favored, transition where it is competition to scavenge the electron, and NO_3_^–^ scavenging favored). The gray dots represent the H_2_O_2_ concentration for pure NaCl droplets at the same concentrations as NaNO_3_ or NaNO_2_. The gray dotted lines represent the standard deviation of the NaCl experiments.

A major difference between the other anions previously studied^7,8^ and NO_2_^–^ and NO_3_^–^ is that those two anions can act as electron scavengers, with rate constants of 3.5 × 10^9^ M^–1^ s^–1^ ^23,24^ and 1 × 10^10^ M^–1^ s^–1^,^25,26^ respectively (reactions [Disp-formula eqR8] and [Disp-formula eqR9] with the oxidation state of nitrogen).

R8 $${{\mathrm{NO}}_{2}}^{-}\left(+\mathrm{III}\right)+e^{-}\to {{\mathrm{NO}}_{2}}^{2-}\left(+\mathrm{II}\right)$$

R9 $${{\mathrm{NO}}_{3}}^{-}\left(+V\right)+e^{-}\to {{\mathrm{NO}}_{3}}^{2-}\left(+\mathrm{IV}\right)$$

These two reactions induce the scavenging of solvated electrons generated via equilibrium (reaction [Disp-formula eqR1]), similar to reactions [Disp-formula eqR3] and [Disp-formula eqR6], which involve oxygen (*k*_O_2__ = 1.9 × 10^10^ M^–1^ s^–1^)^27^ and hydrogen cations (with *k*_H^+^_ = 2.2 × 10^10^ M^–1^ s^–1^)^28,29^ as electron scavengers. However, the reaction between oxygen and electrons (reaction [Disp-formula eqR3]) is the key to the formation of H_2_O_2_ through reactions [Disp-formula eqR3]–[Disp-formula eqR7]. Angelaki et al.^7^ and Mehrgadi et al.^2^ also showed that, in the absence of oxygen, the production of H_2_O_2_ at the interface is negligible. The competition between reactions involving electrons is primarily determined by the concentrations of the reactants within the droplets and their reaction rate constants. Given that these rate constants and concentrations are known, it is possible to estimate which reaction pathway is most likely to be favored in scavenging the produced electron with the branching ratio noted χ from eq 1

1 $$\chi_{\mathrm{scavenger}}\left(\%\right)=\frac{k_{\mathrm{scavenger}}\left[\mathrm{scavenger}\right]}{v_{\mathrm{tot}}}\times 100$$

where [scavenger] is the concentration of the compound that scavenges the electron in M (scavenger = O_2_, H^+^, NO_2_^–^, or NO_3_^–^ depending on the droplet composition), *k*_scavenger_ is the known reaction rate in M^–1^ s^–1^, and *v*_tot_ is the sum of the reaction rate of all the reactions in which electrons are consumed (*v*_tot_ = ∑*k*_scavenger_[scavenger]). The concentration of O_2_ in the droplets is calculated using Henry’s law constant at 293 K (*K*_H_ = 1.3 × 10^–5^ mol m^–3^ Pa^–1^ ^30^), taking into account possible ion-specific salting-out effects as described by the Setchenov relation.^31^ The concentration of H^+^ is calculated based on the pH of the nebulized solution (pH = 6.2 ± 0.5). The value of χ for a scavenger reflects the efficiency with which it reacts with the electron generated at the interface compared to that of the other scavenger. The reaction of the electron with one scavenger can be considered favored when χ_scavenger_ > 90%. If χ_scavenger_ is below 10%, the electron-scavenging efficiency of this scavenger can be considered weak, indicating that this scavenger plays a minor role in the system. When χ_scavenger_ is between 90 and 10%, it can be considered as a transition regime, where the scavenger is in competition with other scavengers. The values of 90 and 10% are arbitrarily chosen to distinguish when the reaction is favored or negligible.

In Figure 2 and Table S2, the electron scavenging efficiencies of these anions are represented by the branching ratio (χ) and compared to that of O_2_. The branching ratio of H^+^ is not represented because its value is around 0.04% throughout the concentration range studied here. The experiments were performed in the transition region, where these two anions compete with O_2_ to scavenge electrons (light-yellow-shaded zone in Figure 2). However, the H_2_O_2_ production by NO_2_^–^/NO_3_^–^ droplets is higher than that by pure NaCl droplets when the NO_3_^–^ or NO_2_^–^ concentration is near to the region where the electron scavenging efficiency is low $\left(\chi_{{{\mathrm{NO}}_{2}}^{-}/{{\mathrm{NO}}_{3}}^{-}}<30\%\right)$ . The enhancement of H_2_O_2_ production by droplets containing anions other than Cl^–^ was already observed. We interpret this change in production due to the propensity of given anions to disrupt the water structure,^32,33^ changing solvation properties at the interface,^4,34^ and, hence, enhance H_2_O_2_ production following the Hofmeister series. To have this effect, the anions need to be at the interface. NO_3_^–^ is somewhat enhanced near the air–water interface,^35–38^ while for NO_2_^–^ ions, no studies are available but a similar behavior might reasonably be expected. As for the other anions already studied,^8^ the presence of NO_2_^–^/NO_3_^–^ at the interface alters the interfacial solvation properties, reducing the ionization energy needed to form OH radicals and the electron from OH^–^.^4^ This may be the reason why, at a low NO_2_^–^/NO_3_^–^ concentration (below 1 mM), H_2_O_2_ production is higher than that of pure NaCl droplets. Experimentally, it is challenging to determine the minimum NO_2_^–^/NO_3_^–^ concentration needed in the droplets to capture the steep changes in the H_2_O_2_ concentration from pure NaCl droplets to droplets containing NO_2_^–^/NO_3_^–^ ions. However, because NO_2_^–^ and NO_3_^–^ can act as electron scavengers, H_2_O_2_ production can be inhibited by reducing the efficiency of reactions [Disp-formula eqR3]–[Disp-formula eqR7]. This is also consistent with studies reporting that, in the absence of oxygen, interfacial production of H_2_O_2_ is negligible.^7,39^ In fact, when the concentration of NO_2_^–^ or NO_3_^–^ increases in the droplets, the relative electron scavenging efficiency of O_2_ decreases in favor of NO_2_^–^ or NO_3_^–^. This results in a reduction in the amount of H_2_O_2_ produced in NO_2_^–^- or NO_3_^–^-containing droplets at higher ion concentrations (above 1 mM).

For NO_3_^–^ droplets (Figure 2A), the amount of H_2_O_2_ produced remains higher than that of pure NaCl droplets in the transition regime, where O_2_ and NO_3_^–^ compete to scavenge electrons (light-yellow-shaded zone in Figure 2). However, when reaction [Disp-formula eqR9] dominates, the H_2_O_2_ concentration produced by NO_3_^–^ droplets falls below that of pure NaCl droplets. The fact that H_2_O_2_ is still detected when O_2_ scavenging is negligible compared to NO_3_^–^ can be due to the fact that the oxygen pathway (reactions [Disp-formula eqR3]–[Disp-formula eqR7]) is not the only way to form H_2_O_2_ from the dissociation of OH^–^ to OH radicals and the electron (reaction [Disp-formula eqR1]); indeed, two OH radicals may recombine as well to form H_2_O_2_ (reaction [Disp-formula eqR2]), and this reaction can proceed even when the electron is scavenged by NO_3_^–^.

The dependence of the H_2_O_2_ concentration on the O_2_ branching ratio is shown in Figure 3. *R* ^2^ of this correlation is 0.80 with a deviation in the regime where O_2_ scavenging efficiency is negligible (χ_O_2__ < 10%), in which the H_2_O_2_ formation can mainly be attributed to reaction [Disp-formula eqR2] only. The branching ratio and H_2_O_2_ measurement confirms that NO_3_^–^ anions act as an electron scavenger and that H_2_O_2_ production is governed by competition between NO_3_^–^ and O_2_ to scavenge the interfacial electrons generated by reaction [Disp-formula eqR1]. However, this explanation does not apply to the case of the NO_2_^–^ droplets (Figure 2B). Indeed, the decrease observed in the H_2_O_2_ concentration produced by NO_2_^–^ droplets is too “fast” compared to the competition between O_2_ and NO_2_^–^ to scavenge the electron (represented by the branching ratio in Figure 2). This suggests the involvement of an alternative chemical pathway^40,41^ in NO_2_^–^ droplets, which inhibits H_2_O_2_ production.

**Figure 3:**
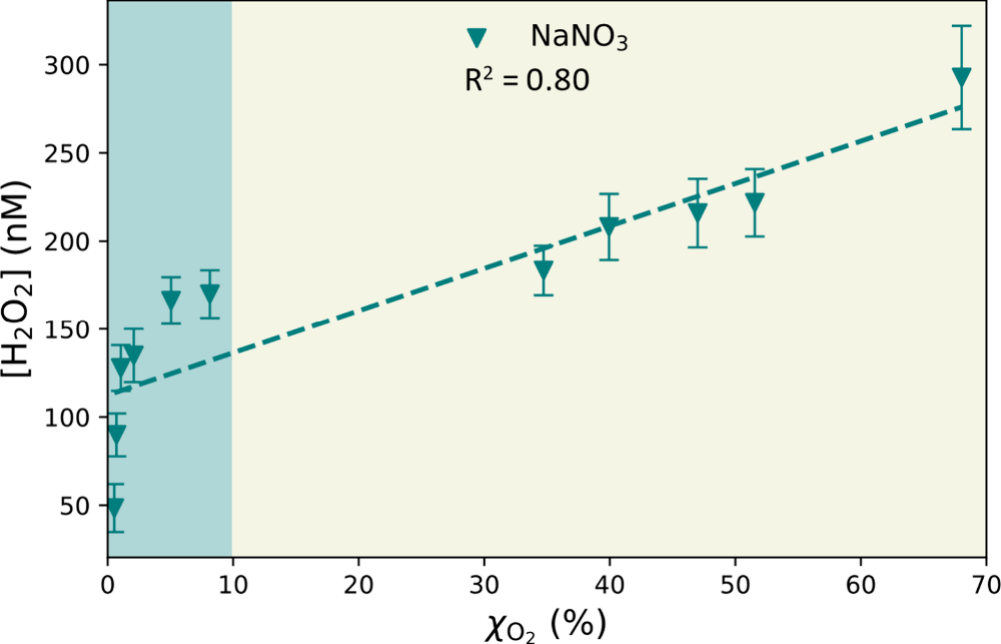
Mean H_2_O_2_ concentration of NaNO_3_ droplets as a function of the corresponding branching ratio of O_2_ (at the concentration of NaNO_3_ used). The dashed line represents a linear fitting of the data.

In all of the experiments presented above, ion chromatographic (IC) analysis was performed on both the solution prior to nebulization and the droplets. For concentrations in excess of 1 mM, solutions were diluted prior to analysis. The characteristic peaks of NO_2_^–^ and NO_3_^–^ as well as their calibration curves used for quantification are available in Figure S4. In the droplets, only the characteristic peaks of NO_3_^–^ and Cl^–^ were detected, suggesting that NO_3_^–^ is reduced by its reaction with the solvated electron, without producing NO_2_^–^. In the case of NO_2_^–^, the detection of trace amounts of NO_3_^–^ in the droplets (in contrast to the bulk solution) suggests spontaneous oxidation of NO_2_^–^. These results are presented in Figure S5 and Table S3. Within the droplets, NO_2_^–^ oxidation could contribute to the rapid suppression of H_2_O_2_ production observed in Figure 1 by its reaction with OH radicals, thus reducing the importance of reaction [Disp-formula eqR2] that forms H_2_O_2_.

The conversion of NO_2_^–^ to NO_3_^–^ has already been observed in single NO_2_^–^ droplets on the molar scale, with a conversion efficiency as high as 80% using an optical tweezer.^40,41^ To explain such observations, we suggest the chemistry described by reactions [Disp-formula eqR10]–[Disp-formula eqR13] below, where NO_2_^–^ acts also as an electron donor (and not just an electron scavenger). Since the droplets are slightly acidic (pH = 6.2 ± 0.5), nitric acid will dissociate into NO_3_^–^ and H^+^ (reaction [Disp-formula eqR13]).

R10 NO2−⁡(+III)⇌NO2•⁡(+IV)+e−

R11 NO2−⁡(+III)+OH•→NO2•⁡(+IV)+OH−

R12 NO2•⁡(+IV)+OH•→HNO3⁡(+V)

R13 $${\mathrm{HNO}}_{3}\left(+V\right)⇌H^{+}+{{\mathrm{NO}}_{3}}^{-}\left(+V\right)$$

The proposed mechanism involves nitrogen dioxide (NO_2_), a gaseous species that can be detected with a NO_*x*_ analyzer. Therefore, additional experiments, including gas-phase measurements, were carried out to investigate this spontaneous oxidation of NO_2_^–^.

### 3.2 Oxidation of NO_2_^–^ in Droplets as a Source of Gaseous NO_2_

To measure NO_2_ with a chemiluminescence NO_*x*_ analyzer, the HONO concentrations have to be kept as low as possible in order to limit interferences. HONO is the acidic form of NO_2_^–^, which means that a fraction of NO_2_^–^ is present in the form of HONO.^42^

R14 $$\mathrm{HONO}⇌H^{+}+{{\mathrm{NO}}_{2}}^{-}$$

Reaction [Disp-formula eqR14] shows the equilibrium between HONO and NO_2_^–^, and the p*K*_a_ at 293 K is 3.0 ± 0.1.^43^ The pH of the NO_2_^–^ solutions used in the previous experiments was 6.2 ± 0.5. At this pH, around 0.1% of NO_2_^–^ protonates to HONO; at equilibrium, this yields around 23 ppb_v_ of HONO from a 1 mM NO_2_^–^ solution. If this amount of HONO is present during the droplet experiments, the determination of the NO_2_ concentration will be compromised by HONO interference in the NO_*x*_ analyzer. To avoid this, the pH of the NaNO_2_ solution was adjusted to 10.5 ± 0.2 with NaOH before nebulization to minimize HONO release from the droplets. Also, during experiments, HONO was monitored using a LOPAP instrument in order to quantify interferences in the NO_2_ signal arising from HONO. The method to correct the NO_2_ concentration is detailed in Text S1, Figures S6–S8, and Table S4. Based on this method, around 9.2% of the HONO concentration measured by the LOPAP is taken as an interference in the NO_2_ signal of the NO_*x*_ analyzer. Gas-phase measurements were performed during experiments where a solution of 1 mM NO_2_^–^ was used, as at this concentration, NO_3_^–^ was detected in the droplets (Figure 3). The experimental duration was kept short (20 min) to avoid equilibrium between the gas phase and the droplets, reducing instrumental contamination by HONO.

Figure 4 shows the NO_*x*_ concentration measured for an experiment where 1 mM NaNO_2_ was sprayed at pH 10.5 (replicated 3 times). The HONO concentration and interferences are presented in Figure S9 and Text S2. During the spraying (in the blue-shaded region), NO and NO_2_ were produced by the droplets. When the spray stopped, both concentrations decreased to zero. This confirms that NO_2_^–^ droplets produced NO_2_ and NO. Another indication of NO_*x*_ production by NO_2_^–^ droplets is that the signal of the interference channel (Figure S10) increases during the nebulizing time. This channel of LOPAP confirms that a species other than HONO is measured during the experiment; the most likely gaseous compound to induce this is NO_2_. The NO_2_ concentration reported in this study is determined after correcting for HONO interference in the NO_*x*_ analyzer. While these analytical methods introduce uncertainties and reduce sensitivity, the production of NO_2_ by NO_2_^–^ containing droplets remains the most plausible explanation for the observed decrease in H_2_O_2_ production (Figure 2). This interpretation is further supported by the oxidation of NO_2_^–^ to NO_3_^–^, as illustrated in Figure S5. At the interface, droplets containing NO_2_^–^ induce an inhibition of the H_2_O_2_ production, concomitant with a NO_2_^–^ oxidation chemistry that produces NO_2_ and NO_3_^–^ with a slight reduction of NO_2_^–^ to NO. NO_2_ and NO_3_^–^ formation can be explained by reactions [Disp-formula eqR10]–[Disp-formula eqR13]. This pathway inhibits H_2_O_2_ production by scavenging OH radicals (limiting reaction [Disp-formula eqR2]) but should also enhance the oxygen pathway (reactions [Disp-formula eqR3]–[Disp-formula eqR7]) due to the dissociation of NO_2_^–^ into NO_2_ and solvated electrons (reaction [Disp-formula eqR10]). The oxygen pathway to produce H_2_O_2_ can be limited by either the electron scavenging of NO_2_^–^ (reaction [Disp-formula eqR8]) or reaction [Disp-formula eqR15]. The relatively small production of NO can be explained by reactions [Disp-formula eqR16] and [Disp-formula eqR17] (see Table S5).

R15 $${\mathrm{NO}}_{2}\left(+\mathrm{IV}\right)+{O_{2}}^{-}\to {{\mathrm{NO}}_{2}}^{-}\left(+\mathrm{III}\right)+O_{2}$$

R16 $${{\mathrm{NO}}_{2}}^{2-}\left(+\mathrm{II}\right)+H_{2}O\to \mathrm{NO}\left(+\mathrm{II}\right)+2{\mathrm{OH}}^{-}$$

R17 $${{\mathrm{NO}}_{2}}^{-}\left(+\mathrm{III}\right)+H^{•}\to \mathrm{NO}\left(+\mathrm{II}\right)+{\mathrm{OH}}^{-}$$

Figure 5 and Table S5 summarize the specific chemistry at the air–water interface of the NO_2_^–^ droplets.

**Figure 4:**
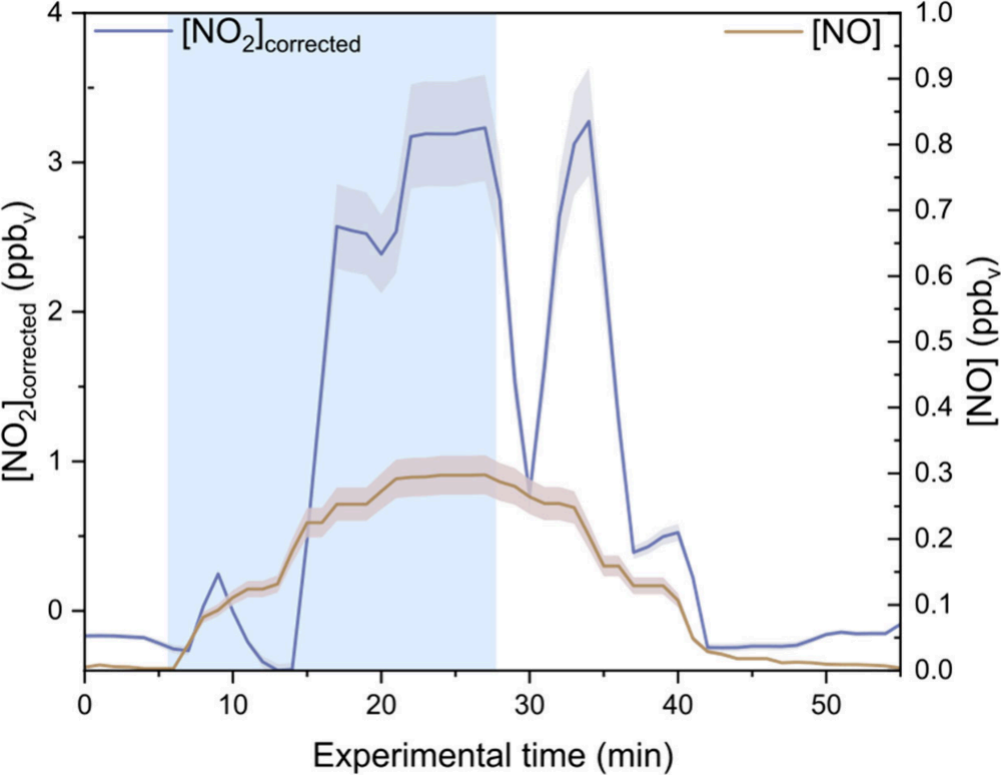
NO_2_ and NO measurement for droplets containing 1 mM NaNO_2_ at pH 10.5. The blue shading represents the time at which droplets are generated in the reactor. The NO_2_ concentration is corrected from HONO interference. The shadings around NO and NO_2_ concentrations represent the experimental uncertainties. The drop in the corrected NO_2_ concentration at 10 and 27 min is mainly due to the time response correction of the two instruments. The temporal evolution of the concentrations observed does not reflect the actual chemical kinetics of the studied system but is rather governed by the instrumental response time.

**Figure 5:**
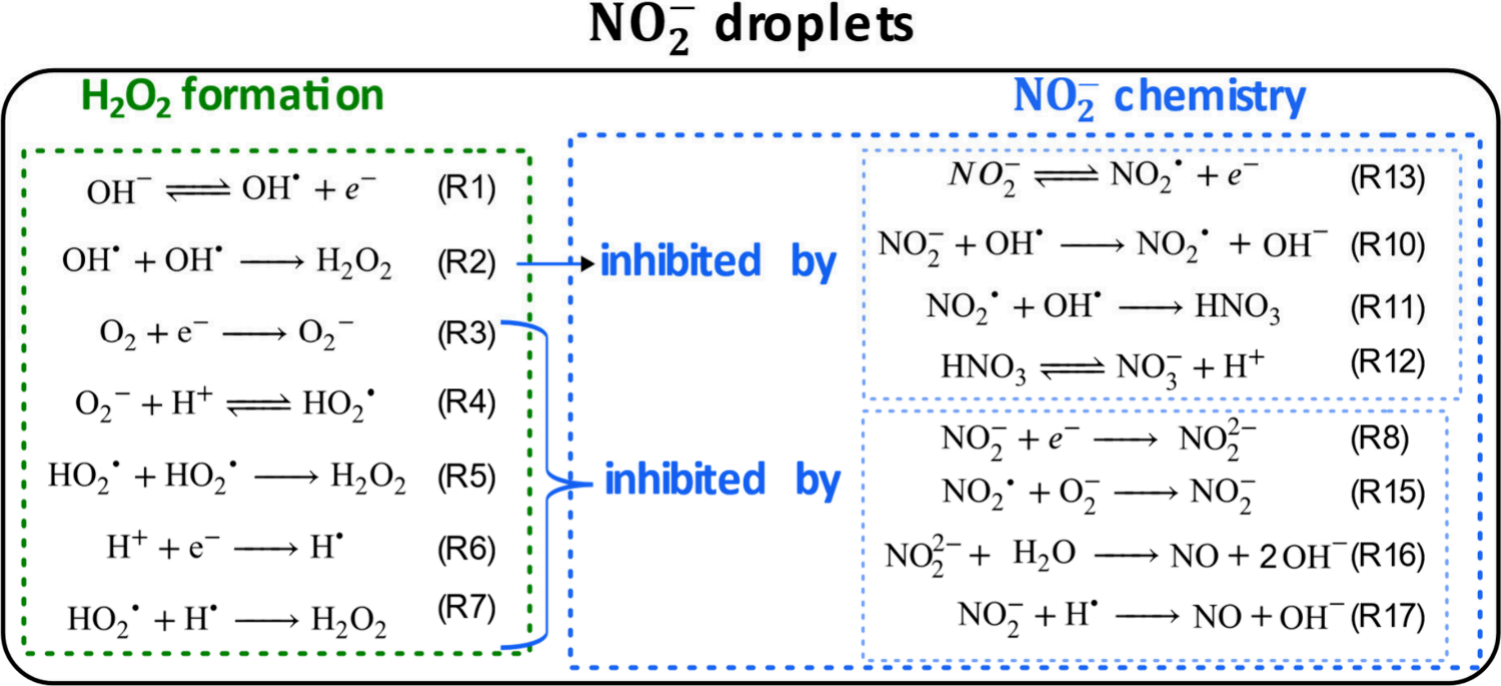
Schematic of the proposed interfacial mechanism that occurs in NO_2_^–^ droplets under dark conditions.

### 3.3 Spontaneous HONO Production by NO_3_^–^ Droplets under Dark Conditions

Since NO_3_^–^ exhibits the same capacity, though to a lesser extent, as NO_2_^–^ to reduce H_2_O_2_ production at the air–water interface (Figure 2A), we explored whether the nitrate anion could also trigger additional chemistry, eventually leading to the formation of NO, NO_2_, or HONO as well. In contrast to the nitrite anion case, a higher concentration (i.e., above 1 mM as no instrumental interference was evidenced) and near neutral conditions could be used. Figure 6 shows the HONO concentration measured from spraying of droplets containing 100 to 1000 mM NaNO_3_; below this concentration, no HONO was detected. During the experiments, NO and NO_2_ signals were below the limit of detection (LOD = 50 ppt_v_) of the NO_*x*_ analyzer and are presented in Figure S11.

**Figure 6:**
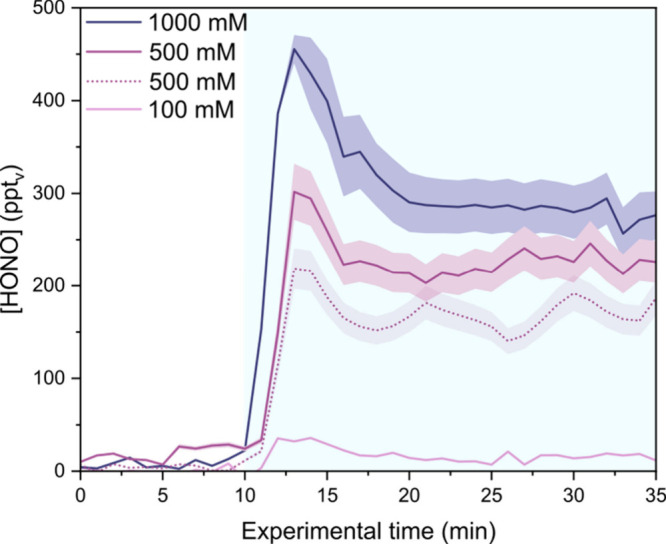
HONO concentration measured in droplets containing *x* mM NaNO_3_ at pH 6.2. The dotted line represents experiments performed with the addition of 10 mM NaSCN in the droplets. The blue shading represents the spraying time. The shadings represent the standard deviations of the experiments. The temporal evolution of the concentrations observed does not reflect the actual chemical kinetics of the studied system but is rather governed by the instrumental response time.

Unexpectedly, when NO_3_^–^-containing droplets are in the reactor, ppt_v_ levels of HONO are detected under dark conditions. At 100 mM NO_3_^–^, measured HONO is close to the LOD of the instrument (20 ppt_v_), but the signal increases at higher concentration. At these concentrations, NO_3_^–^ acts as the sole electron scavenger (based on the branching ratio of Figure 2). If HONO is detected at this pH, then it is possible that NO_2_^–^ is present in the droplets. Unfortunately, IC measurements could not confirm the formation of traces of NO_2_^–^ in the droplets (note that solutions are diluted by a factor of 1000 as the initial NO_3_^–^ levels are too high to be injected directly in the IC). With this dilution, if trace NO_2_^–^ is produced by the droplets, the concentration in the IC sample can easily go below the LOD. To explore in more real-life atmospheric conditions this chemistry of NO_3_^–^ droplets, experiments were performed in an atmospheric chamber (Figure S12), in which the reaction time is significantly longer (1 h and 40 min in the chamber compared to 20 s in the flow tube reactor), which could lead to higher HONO production and possibly the detection of NO_2_ or NO. The results of these experiments are shown in Figure S13. Higher amounts of HONO and NO_*x*_ were observed, but both NO_2_ and NO signals were still near the LOD of the instrument (and hence uncertain). In atmospheric simulation chambers, wall loses are very important,^44,45^ explaining partly why the observed HONO concentration does not scale linearly between the two experimental setups and timescales.^46,47^ To investigate whether OH radicals play a role in NO_3_^–^ reduction, 10 mM NaSCN was added to the NaNO_3_ solution before nebulization in some experiments (dotted line in Figure 6). SCN^–^ reacts with OH with a rate constant of 1.4 × 10^10^ M^–1^ s^–1^.^48,49^ During these experiments, HONO can still be measured but at lower levels compared to droplets containing solely NO_3_^–^. The addition of SCN^–^ reduces HONO production by only ca. 30%, suggesting that OH radicals are not only involved in HONO production in NO_3_^–^ droplets. In fact, following the electron scavenging by nitrate anions, a series of chemical reactions may occur, as described by the following mechanism:

$${{\mathrm{NO}}_{3}}^{-}\left(+V\right)+e^{-}\to {{\mathrm{NO}}_{3}}^{2-}\left(+\mathrm{IV}\right),\left(R9\right)$$

R18 $${{\mathrm{NO}}_{3}}^{2-}\left(+\mathrm{IV}\right)+O_{2}\to {{\mathrm{NO}}_{3}}^{-}\left(+V\right)+{O_{2}}^{-}$$

R19 $${{\mathrm{NO}}_{3}}^{2-}\left(+\mathrm{IV}\right)+H^{+}\to {\mathrm{NO}}_{2}\left(+\mathrm{IV}\right)+{\mathrm{OH}}^{-}$$

$$H^{+}+e^{-}\to H^{•},\left(R6\right)$$

R20 $${\mathrm{NO}}_{2}\left(+\mathrm{IV}\right)+H^{•}\to \mathrm{HONO}\left(+\mathrm{III}\right)$$

R21 $${{\mathrm{NO}}_{3}}^{2-}\left(+\mathrm{IV}\right)+{\mathrm{NO}}_{2}\left(+\mathrm{IV}\right)\to {{\mathrm{NO}}_{3}}^{-}\left(+V\right)+{{\mathrm{NO}}_{2}}^{-}\left(+\mathrm{III}\right)$$

$${\mathrm{NO}}_{2}\left(+\mathrm{IV}\right)+{O_{2}}^{-}\to {{\mathrm{NO}}_{2}}^{-}\left(+\mathrm{III}\right)+O_{2},\left(R15\right)$$

$$H^{+}+{{\mathrm{NO}}_{2}}^{-}\left(+\mathrm{III}\right)⇌\mathrm{HONO}\left(+\mathrm{III}\right),\left(R14\right)$$

With this mechanistic framework, HONO can form via nitrite (NO_2_^–^) generated by reactions [Disp-formula eqR21] and [Disp-formula eqR15]. However, owing to instrumental limitations, NO_2_^–^ production by NO_3_^–^ droplets could not be confirmed. The formation of HONO (and thus NO_2_^–^) most likely requires NO_2_, yet NO_2_ was not detected in any of the NO_3_^–^ droplet experiments. Reactions involving NO_2_ nevertheless remain plausible because HONO concentrations are low; any co-produced NO_2_ would likely be below the detection limit of the NO_*x*_ analyzer (50 ppt_v_). Notably, the proposed mechanism does not account for the decrease in the level of HONO when droplets contain an OH scavenger. This point warrants further investigation to clarify NO_3_^–^ interfacial chemistry. Although the mechanism remains unresolved, NO_3_^–^ droplets do produce HONO. In clouds, the air-equivalent concentration of nitrate (NO_3_^–^) typically^50^ ranges from 0.1 to a few μg m^–3^. This concentration is much smaller than the concentration used in this study. However, in atmospheric aerosols, molar levels of NO_3_^–^ are possible,^51^ suggesting that NO_3_^–^ droplets may be an important source of HONO at night, in contrast to the day time, when nitrate particles undergo atmospheric photolysis.^21^

In atmospheric aerosols, ammonium nitrate (NH_4_NO_3_)^52,53^ predominates over sodium nitrate (NaNO_3_). To investigate the chemistry under more atmospherically relevant conditions, experiments with 500 mM NH_4_NO_3_ were also performed in the atmospheric simulation chamber (Figure S12). In contrast to NaNO_3_ droplets, no HONO is detected from NH_4_NO_3_ droplets (Figure S14). This suggests that the NO_3_^–^ chemistry is cation-dependent and needs more investigation to understand the mechanism. A possible explanation for this cation dependence is a reaction between NH_4_^+^ and NO_3_^2–^ (i.e., reaction [Disp-formula eqR22])­

R22 $${{\mathrm{NH}}_{4}}^{+}+{{\mathrm{NO}}_{3}}^{2-}⇌{\mathrm{NH}}_{3}+{\mathrm{OH}}^{-}+{\mathrm{NO}}_{2}$$

which proceeds with a rate constant of 2 × 10^8^ M^–1^ s^–1^.^54^ This reaction introduces a specificity between NH_4_NO_3_- and NaNO_3_-containing droplets, indirectly confirming that HONO production is linked to NO_3_^2–^ arising from the reduction of NO_3_^–^ at the interface (reaction [Disp-formula eqR8]). A second possibility is that reaction [Disp-formula eqR22] increases the pH of the droplets shifting the equilibrium NO_2_^–^/HONO with less HONO partitioning to the gas phase. Figure S15 and Table S6 summarize all of the reactions proposed in the case of NO_3_^–^ droplets.

At the air–water interface, dependent upon the concentration, NO_2_^–^ and NO_3_^–^ showed unexpected dark reactivity that inhibits H_2_O_2_ spontaneous formation. At a low concentration (below 0.6 mM), NO_2_^–^ and NO_3_^–^ produced more H_2_O_2_ compared to NaCl droplets due to their ability to disrupt the structure of the water at the interface.^8^ When the concentrations of NO_2_^–^ or NO_3_^–^ increased in the droplets, H_2_O_2_ production is finally inhibited. Although both anions exhibit this specificity, their mechanisms differ. In the case of NO_2_^–^, competition between H_2_O_2_ formation and spontaneous NO_2_^–^ oxidation explains this H_2_O_2_ inhibition. This is confirmed by detection of NO_3_^–^ in the droplets and NO_2_ in the gas phase when a solution of NaNO_2_ is nebulized. This unexpected chemistry at the interface may provide another NO_2_ source at night time. NO_3_^–^ chemistry at the air–water interface is less clear. The H_2_O_2_ inhibition in NO_3_^–^ droplets can be explained by the fact that NO_3_^–^ acts as electron scavenger like O_2_. The decrease in H_2_O_2_ production with the NO_3_^–^ concentration fairly well follows the shift in electron scavenging from dissolved O_2_ to NO_3_^–^. This finding confirms that, in the droplets, the reactivity at the interface is governed by the compound with the higher efficiency to scavenge the electron. We propose that the reaction between NO_3_^–^ and the electron may initiate specific NO_3_^–^ chemistry under dark conditions that is similar to nitrate photolysis. However, only HONO is detected at the ppt_v_ level for droplets containing a molar level of NO_3_^–^. The detection of HONO is unexpected and not fully understood, but it suggests a new chemistry of NO_3_^–^ at the air–water interface that does not require any light or catalysis. Simulations and further experiments are needed to fully understand nitrate chemistry at the interface after scavenging the electron.

## Supplementary Material



## Notes

Size distribution (Figure S1), bulk H_2_O_2_ measurement (Figure S2), H_2_O_2_ concentration as a function of ion’s activities (Figure S3), experimental data (Table S1), value of the calculated branching ratio (Table S2), ion chromatography calibration curve (Figure S4), ion chromatography results (Figure S5), IC data (Table S3), quantification of HONO interference in the NO_*x*_ analyzer (text S1, Figures S6–S8, and Table S4), HONO data (Figure S9), role of CO_2_ in the HONO concentration measured (text S2), signal of the interferent channel of the LOPAP (Figure S10), proposed mechanistic pathways for NO_2_^–^ droplets (Table S5), NO_*x*_ measurement for NO_3_^–^ droplets (Figure S11), atmospheric chamber setup (Figure S12), gas-phase measurement in the atmospheric chamber (Figure S13), comparison between NH_4_NO_3_ and NaNO_3_ droplets (Figure S14), proposed mechanistic pathways for NO_3_^–^ droplets (Figure S15), and proposed mechanistic pathways for NO_3_^–^ droplets (Table S6) (PDF)
