# Supplementary material for: Specific Ion Chemistry at the Air–Water Interface of Nitrite/Nitrate-Containing Droplets
Source: Environ Sci Technol. 2026 Mar 20;60(15):11577–85. doi: 10.1021/acs.est.5c15074 (PMC13104025; doi:10.1021/acs.est.5c15074)
Supplement: Supplementary file 1 [file es5c15074_si_001.pdf]

Specific ion chemistry at the air-water interface of nitrite/nitrate containing droplets

Yoan Carreira Mendes Da Silva[1], Maria Angelaki[1], Adrien Gandolfo[1], D. James Donaldson[2,3] and Christian George*[,1]

[1]Universite Claude Bernard Lyon 1, CNRS, IRCELYON, UMR 5256, Villeurbanne, F-69100, France

[2]Department of Chemistry, University of Toronto, 80 George Street, Toronto, Ontario, Canada M5S 3H6

[3]Department of Physical and Environmental Sciences, University of Toronto, Scarborough, 1265 Military Trail, Toronto, ON Canada M1C 1A4

*To whom correspondence should be addressed: christian.george@ircelyon.univ-lyon1.fr

Number of pages: 20

Number of tables: 6

Number of figures: 15

Supporting Information

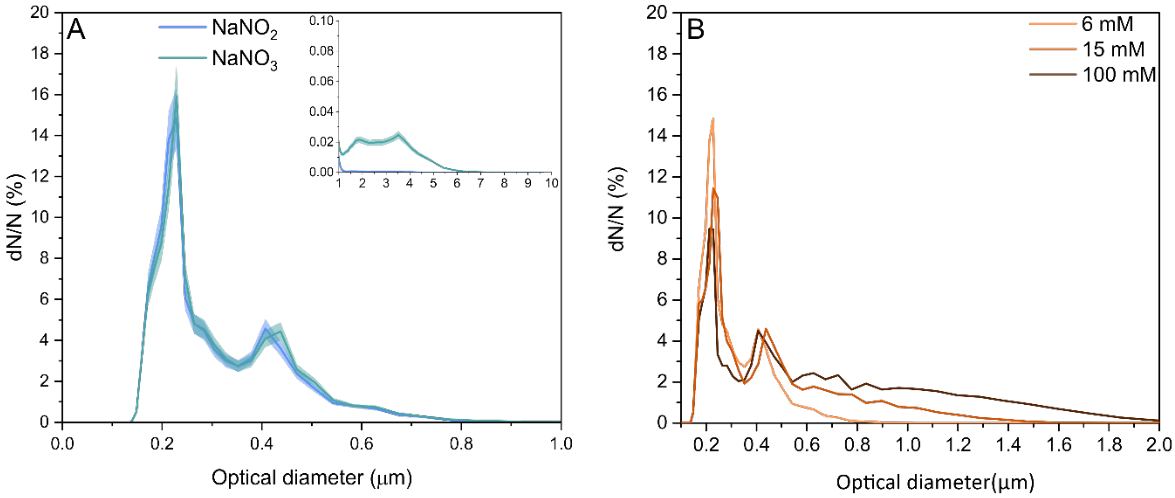

Figure S1. Normalized size distribution of droplets containing 6 mM $NaNO_2$ or $NaNO_3$ measured using an OPC. The inset shows a zoom of droplet diameters above 1 μm. The shaded areas represent the experimental uncertainty. B) Normalized size distribution of droplets containing x mM of $NaNO_3$ measured using an OPC. Direct measurements at concentrations higher than 100 mM could not be performed due to the high droplet number concentration, which exceeds the OPC detection limit. Increasing the solution concentration does not change the overall shape of the size distribution but increases the fraction of larger droplets (diameter > 0.5 μm).

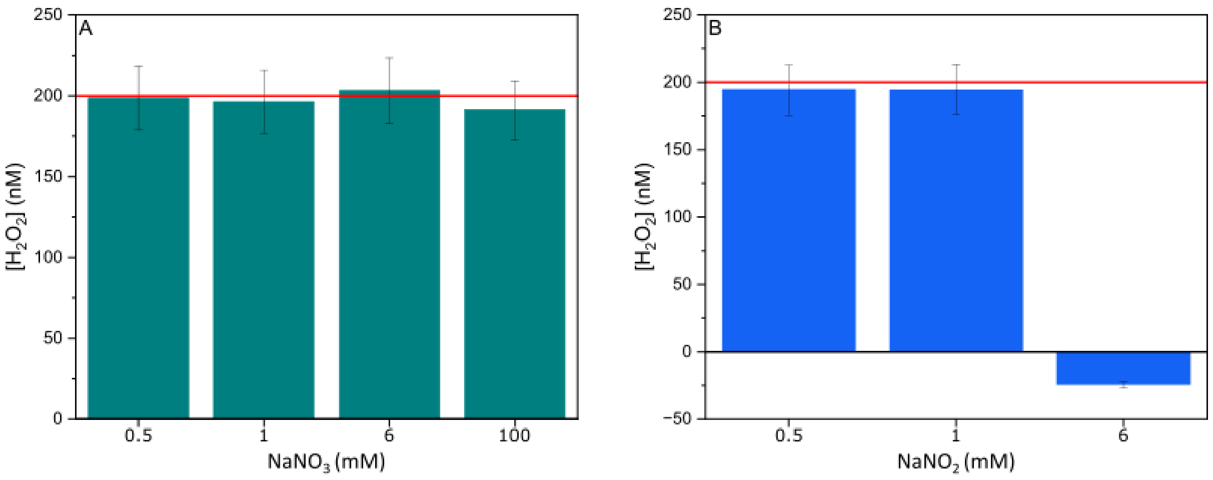

Figure S2. Bulk H_2O_2 measurement of x mM of A) $NaNO_3$ or B) $NaNO_2$ with 200nM of H_2O_2 (red line). When [$NaNO_2$]> 1 mM an interference with the chemical compounds of the

H_2O_2 analyzer can be observed resulting in an impossibility to measure H_2O_2 in solution containing more than 1 mM $NaNO_2$.

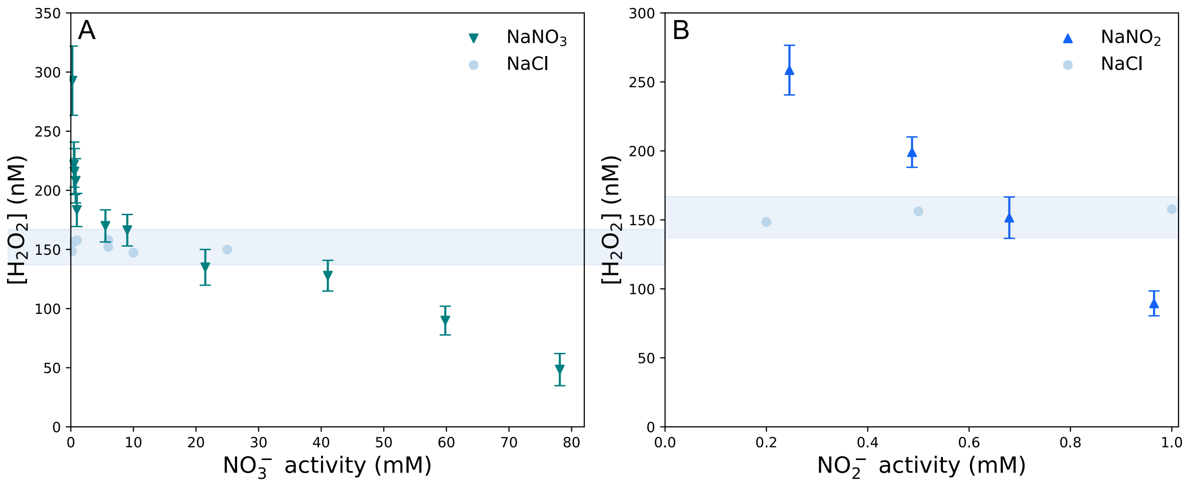

Figure S3. A) H_2O_2 concentration produced by droplets containing x mM of $NaNO_3$ as function of NO_3^- activity. B) H_2O_2 concentration produced by droplets containing x mM of $NaNO_2$ as function of NO_2^- activity. The activity is calculated using Davies equation.[1]

Table S1. H_2O_2 production from droplets containing 6 mM of NaCl with $NaNO_2$ or $NaNO_3$.

Type of ion	[a]$[NO_x^-]$ (mM)	[b]$[H_2O_2] \pm 2\sigma$ (nM)	[c]$[H_2O_2]_{mean} \pm \sigma_{std}$ (nM)
	0.25	271.3 ± 27.2	258.6 ± 18.0
	0.25	245.9± 24.6	
	0.5	206.3 ± 20.7	191.1 ± 16.2
	0.5	192.0 ± 19.3	
	0.7	151.6 ± 15.2	151.6 ± 15.2
	1	85.0 ± 8.2	
NO_2^-	1	94.2 ± 9.5	79.3 ± 12.8
	1	72.4 ± 8.3	
	1	65.7 ± 9.8	
	0.25	292.7 ± 28.3	292.7 ± 28.3
	0.5	229.2 ± 22.3	221.7 ± 22.2
	0.5	214.2 ± 21.5	
	0.6	215.9 ± 21.2	215.9 ± 21.2
	0.8	200.7 ± 20.1	208.1 ± 20.1
	0.8	215.4 ± 21.6	
	1	180.4 ± 18.1	183.4 ± 18.1
	1	186.3 ± 18.7	
NO_3^-	6	169.9 ± 17.0	169.9 ± 17.0
	10	166.3 ± 16.7	166.3 ± 16.7
	25	124.2 ± 12.5	134.9 ± 15.2
	25	145.6 ± 14.6	
	50	127.8 ± 12.8	127.8 ± 12.8
	75	98.4 ± 9.9	89.9 ± 8.9
	75	81.3 ± 8.2	
	100	38.5 ± 5.5	48.4 ± 8.2
	100	58.0 ± 5.1	

[a] Concentration of $NaNO_2$/$NaNO_3$ in the mixture [b] H_2O_2 production measured from droplets and includes systematic uncertainties; [c] mean H_2O_2 concentration with the standard deviation and systematic uncertainties.

Table S2. Branching ratio (χ) for reacting with the electron in the case of NO_2^- and NO_3^- droplets. When $\chi > 90\%$ the reaction is favoured otherwise it is a competition. The branching ratio of H^+ is lower than 0.001% and is not represented in this table.

Type of droplets	$[NO_x^-]$(mM)	χ_{O_2}(%)	$\chi_{NO_x^-}$(%)	Favored reaction
NO_2^- droplets	0.25	85.88	14.12	Competition
	0.5	75.25	24.75	Competition
	0.7	68.47	31.53	Competition
	1	60.32	39.68	Competition
NO_3^- droplets	0.25	68.03	31.97	Competition
	0.5	51.55	48.45	Competition
	0.6	47.00	53.00	Competition
	0.8	39.94	60.06	Competition
	1	34.73	65.27	Competition
	6	8.14	91.86	$NO_3^- + e^- \rightarrow NO_3^{2-}$
	10	5.05	94.95	$NO_3^- + e^- \rightarrow NO_3^{2-}$
	25	2.08	97.92	$NO_3^- + e^- \rightarrow NO_3^{2-}$
	50	1.05	98.95	$NO_3^- + e^- \rightarrow NO_3^{2-}$
	75	0.70	99.30	$NO_3^- + e^- \rightarrow NO_3^{2-}$
	100	0.53	99.47	$NO_3^- + e^- \rightarrow NO_3^{2-}$

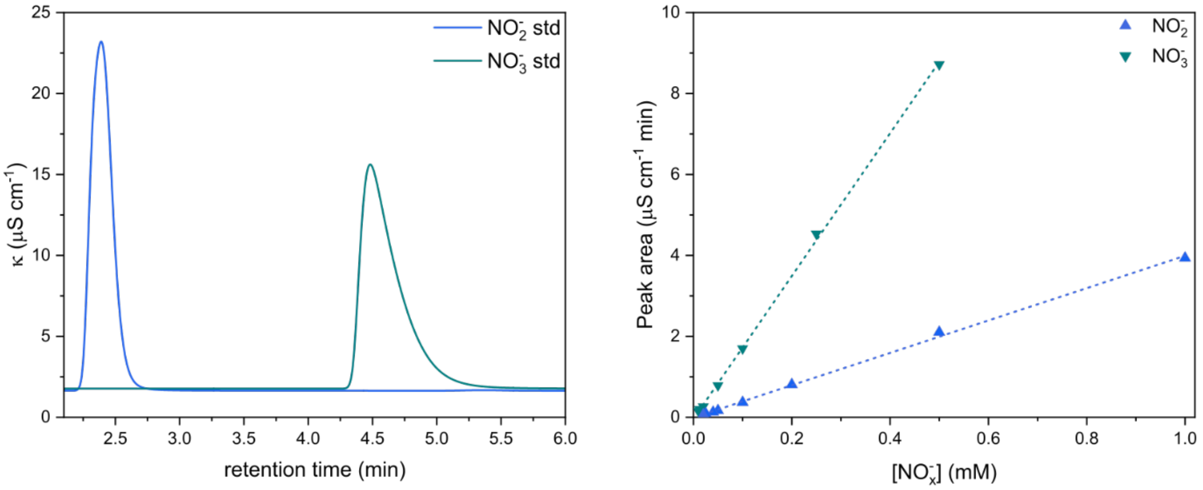

Figure S4. Ion chromatography (IC) of sodium nitrate and sodium nitrite. A) Representative IC chromatogram showing the characteristic peaks of NO_2^- and NO_3^- at 1 mM. B) Calibration curves of NO_2^-, and NO_3^- .

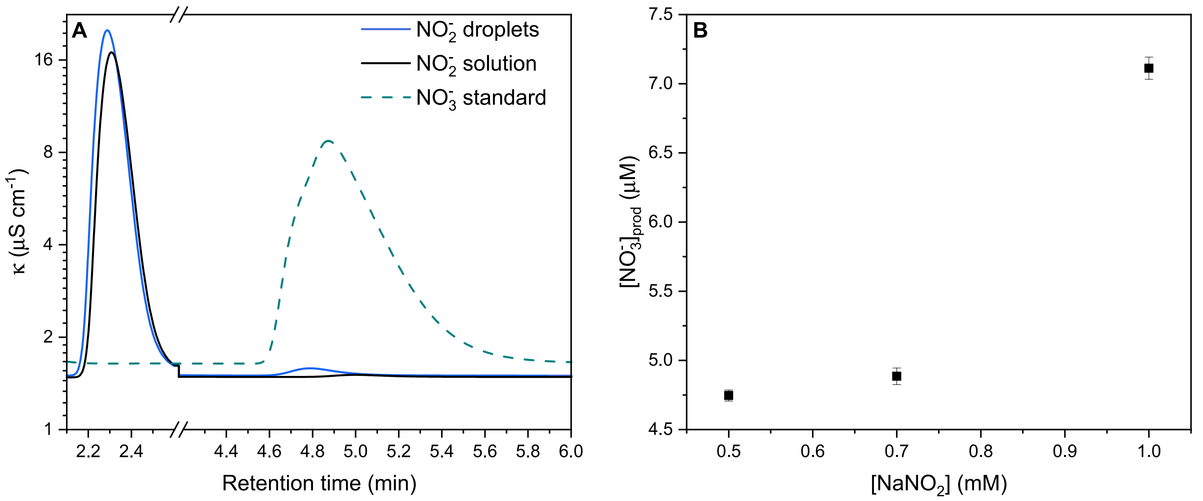

Figure S5: A) Ion chromatogram of one experiment of 1mM $NaNO_2$. Black solid line: $NaNO_2$ bulk solution used for nebulisation, blue solid line: droplets, and green dotted line: NO_3^- standard. B) NO_3^- production observed in droplets containing x mM NO_2^- (no NO_3^- detected in the pre-nebulized solution).

Table S3. Quantification of detected NO_3^- peaks by IC from droplets containing x mM of NO_2^-

$[NO_2^-]$ (mM)	[a]$[NO_3^-]^{IC} \pm 2\sigma$ (µM)	[b]$[NO_3^-]_{mean} \pm \sigma_{std}$ (µM)
0.25	not detected	-
0.25	not detected	
0.5	4.75 ± 0.08	4.75 ± 0.08
0.5	4.74 ± 0.08	
0.7	4.89 ± 0.08	4.89 ± 0.08
1	7.12 ± 0.10	7.46 ± 0.10
1	7.79 ± 0.10	

[a]NO_3^- concentration calculated with calibration curve from Figure S3; [b]mean NO_3^- concentration produced by NO_2^- droplets.

Text S1: Quantification of HONO interference in the NO_x analyzer

To quantify how much HONO interferes with the NO_x analyzer, an additional setup was used and can be seen in Figure S6. An x mM $NaNO_2$ solution at an alkaline pH (11.4 ± 0.1) was injected into a glass bottle where small air flow transfers any gas phase products from the solution to the LOPAP and NO_x analyzer. Since this flow is not sufficient to supply both instruments, an additional humidified flow (RH = 80 %) is added, with an exhaust. Under these conditions, a fraction of the NO_2^- solution is present as HONO, which partitions directly into the gas phase.[2] In principle, whatever the concentration of NO_2^-, only the LOPAP should detect HONO and the NO_x analyzer should not detect NO or NO_2.

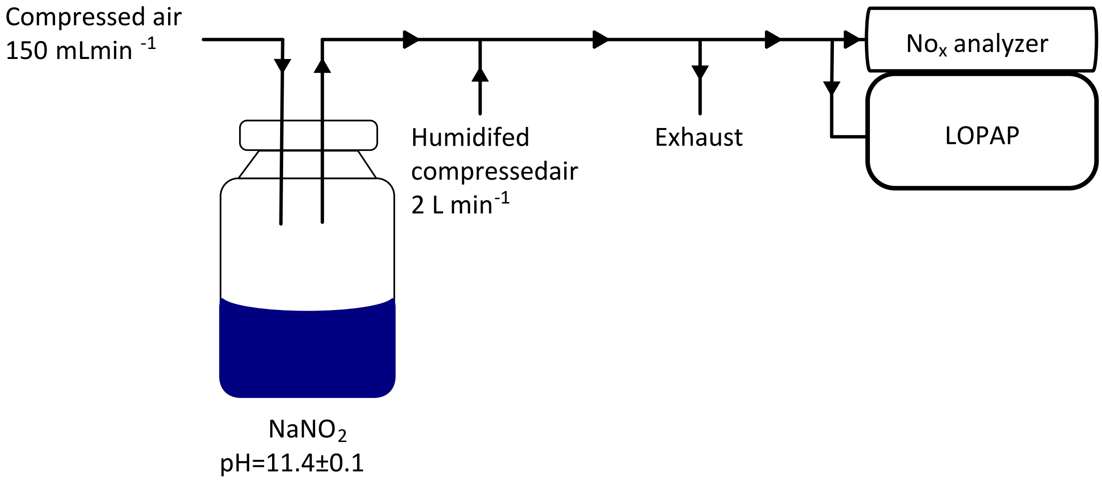

Figure S6: Experimental setup used to quantify the interference of HONO in the NO_x analyzer.

This setup was used with four different concentrations of $NaNO_2$ with always the same pH (adjusted with NaOH). The results of these experiments are presented in Figure S7. HONO, NO and NO_2 were measured. The trends of both instruments are similar, with an increase after a few minutes of sampling and then a reduction of the signal until stabilization. The observed maxima are characteristic of the instrument response from pure air to the air interacting with the solution. These results confirm that HONO is an interference in the NO_x analyzer. The variation between compressed air and the experiments for the NO channel is around 0.01 ppb, which is the noise level of the instrument. However, for the NO_2 channel the variation between air and the experiments can reach 1 ppb (Figure S7D) in the range of $NaNO_2$ tested.

In Figure S7, the HONO concentration measured by the LOPAP is a few ppb. However at the pH of the NO_2^- solution (pH=11.4), HONO should be negligible[2,3], and not this high . Note that the air used here is highly purified compressed air containing CO_2, which gradually acidifies the solution, thereby enhancing HONO degassing. This could explain the HONO concentration in Figure S7. Unfortunately, the pH of the solution was not measured after the experiments to confirm this.

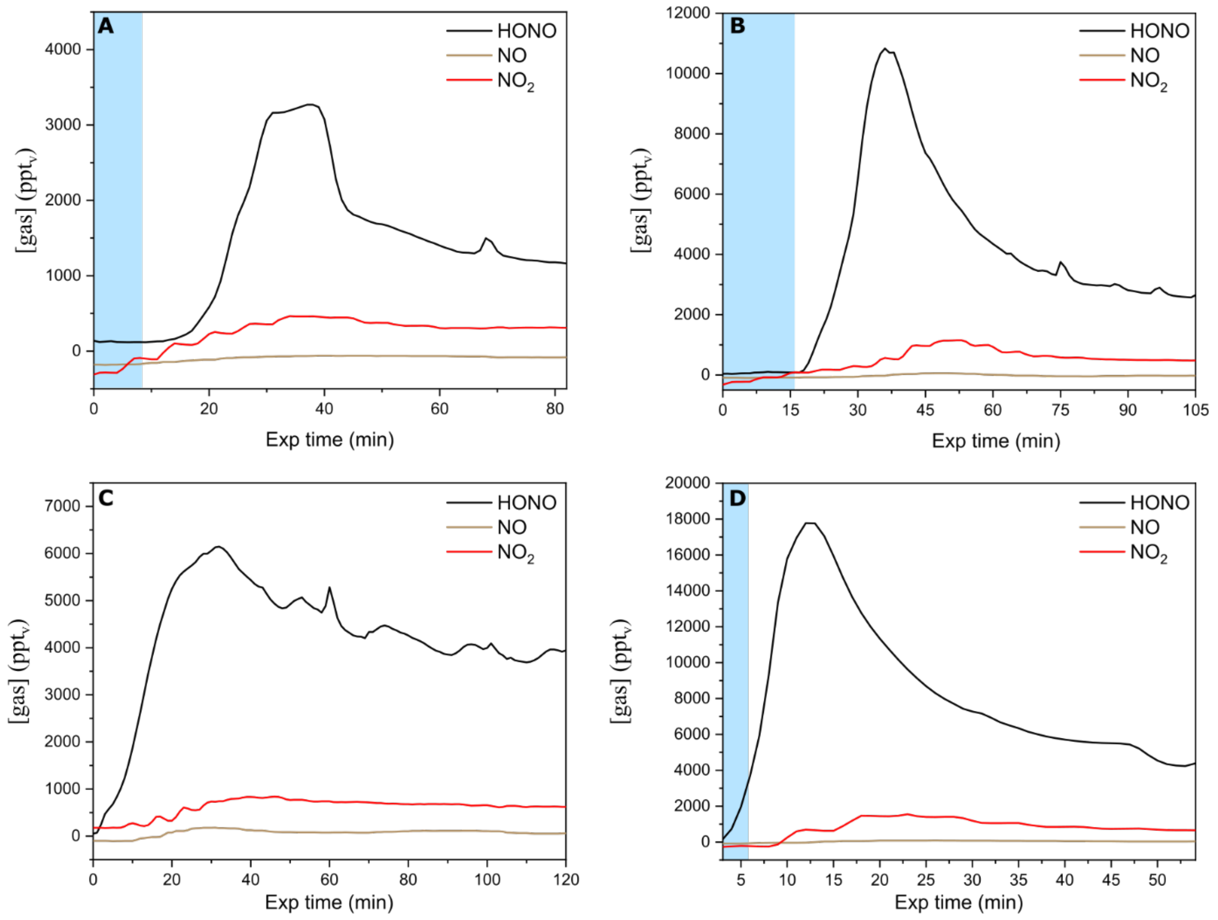

Figure S7: HONO, NO and NO_2 concentration from solution of x mM $NaNO_2$ at pH 11.4. HONO concentration is measured with the LOPAP and the NO_x by the NO_x analyzer. The blue shading represents compressed air before injection of the solution. A) results at 1 mM, B) results at 25 mM, C) results at 50 mM and D) results at 240 mM.

In these bulk experiments only HONO should be detected. That is why the trend observed in the NO_2 channel of the NO_x analyzer is only a HONO interference. Using the measured HONO concentration with the LOPAP, it should be possible to reproduce the NO_2 channel of the NO_x analyzer by solving Equation S1

$$[NO_2]^{NOx}_{measured}(t) = a\,[HONO]^{LOPAP}_{measured}(t) + b \qquad \text{(Eq S1)}$$

where $[NO_2]^{NOx}_{measured}$ is the NO_2 concentration measured by the NO_x analyzer in ppt_v, $[HONO]^{LOPAP}_{measured}$ is the HONO concentration measured by the LOPAP in ppt_v and a and b are the slope and intercept of the fitting, respectively. This equation is valid only because in

this bulk experiment only HONO should be detected and that the signal on the NO_x analyzer is solely due to the HONO interference. Using a Python code, the slope and intercept were calculated for the four concentrations performed in Figure S8.

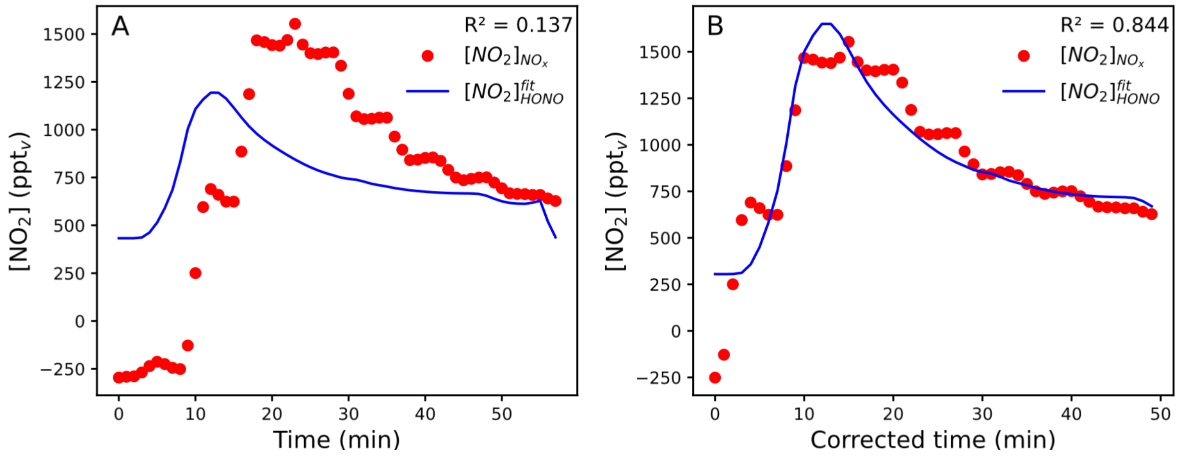

Figure S8: Concentration of NO_2 measured by the NO_x analyzer (red dot) and the "reproduced" NO_2 concentration using HONO concentration measured by the LOPAP and Equation S1 (blue line) for the experiment containing 240 mM of $NaNO_2$. A) using the raw data of each instrument and B) using the data corrected by their instrumental time response.

Figure S8A shows the result of the fit (blue line) when using the raw data of the LOPAP and NO_x analyzer. It is clear that the fit shows poor correlation with the measured data ($R^2 =$ 0.137) ; This can be attributed to the time response of each instrument. To confirm this hypothesis, a correction was applied. Since both instruments have the same sampling line and both detect HONO, one way to correct the time response is to match their maxima. Figure S8B shows the result with this correction, and the agreement between the fit and the measured data is substantially improved. (R^2 = 0.844). The same procedure was performed for all concentrations, the value of the slope (a) and intercept (b) are summarized in Table S4. The mean value of a and b are used to quantify HONO interference in the NO_x analyzer.

Table S4: Fit parameters calculated from HONO data for the four concentrations tested.

[NaNO2] (mM)	slope from the fit (noted a in %)	intercept from the fit (noted b in ppb$_v$)
1	10.8	131.5
25	9.3	298.3
50	8.9	232.5
240	7.6	301.8
**Mean**	**9.2 ± 1.4**	**241.0 ± 79.7**

Based on these results, the NO_2 concentration from HONO interference is calculated by Equation S2 and the real NO_2 concentration by Equation S3:

$$[NO_2]^{HONO}_{interference}(t) = 0.092\,[HONO]^{LOPAP}_{measured}(t) + 241 \quad \text{(Eq S2)}$$

$$[NO_2]_{corrected}(t) = [NO_2]^{NOx}_{measured}(t) - [NO_2]^{HONO}_{interference}(t) \quad \text{(Eq S3)}$$

Note that Equations S2 and S3 are valid only if HONO is detected by the LOPAP; otherwise, Equation S2 loses its physical meaning and introduces an artificial offset (value of the intercept) in the NO_2 concentration calculated using Equation S3. However, including the intercept improves the fit; therefore, the intercept is retained to quantify HONO interference in the NO_x analyzer since this fitting is made for system where HONO is present.

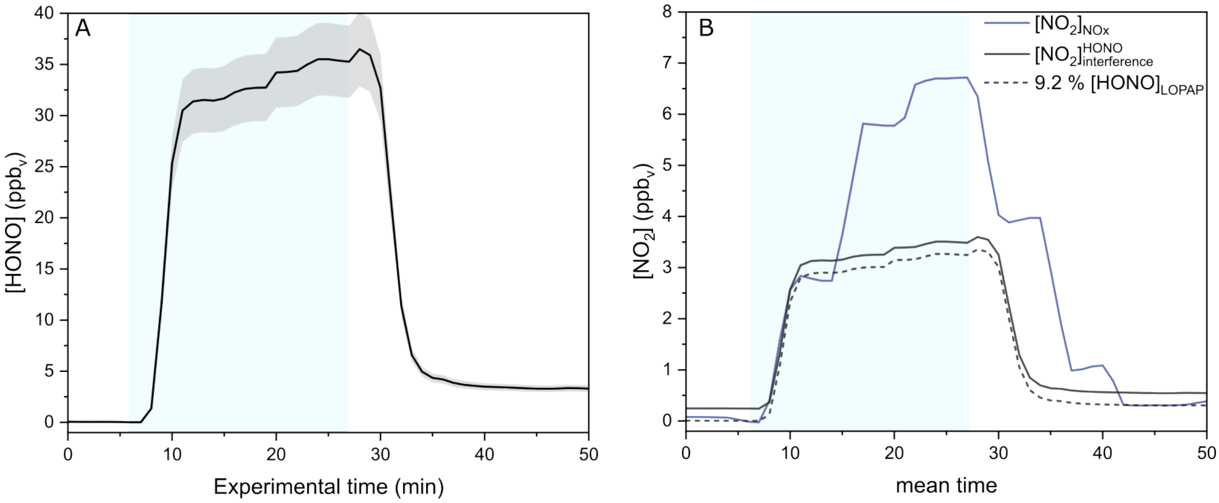

Figure S9: A) HONO concentration measured for droplets containing 1 mM $NaNO_2$ at pH 10.5. B) NO_2 concentration measured by the NO_x analyzer and NO_2 concentration due to HONO interference using Equation S2. The spraying time is represented by the blue shaded region.

Text S2: HONO concentration measured in droplets experiments.

During the 20 min of the experiment, 40 ppb of HONO is released by the droplets at the pH of the nebulized solution (pH=10.5), while HONO should be in principle negligible. This suggests that the pH of the droplets may be more acidic than the solution. The experiments presented here were performed with high purified air containing ambient levels of CO_2. Study on levitated droplets has shown that under alkaline condition the pH of the droplets decrease due to CO_2 uptake.[4] In our group, it was also observed that the pH of collected droplets are lower than the bulk solutions used for nebulisation under alkaline conditions and in presence of CO_2.[5] Under acidic and neutral conditions, the change in pH due to gaseous CO_2 uptake is not observed.[5] Since the experiments are performed under alkaline condition, CO_2 dissolves in the droplets and decreases the pH. This could partly explain why, during spraying, the HONO concentration continues to increase (Figure S9). At the end of the spraying, HONO concentration exceeds the pre-spray baseline (by 3.3 ± 0.4 ppb_v) due to the presence of aqueous NO_2^- from droplets impaction inside the reactor. The equilibrium between NO_2^- solution at pH 10.5 and HONO under air flow containing CO_2 has been measured with the setup described in Figure S6. HONO concentration in this equilibrium experiment was 3.5 ± 0.4 ppb_v confirming that HONO concentration at the end of the droplets experiment (Figure S9A) is due to the equilibrium HONO/NO_2^-.

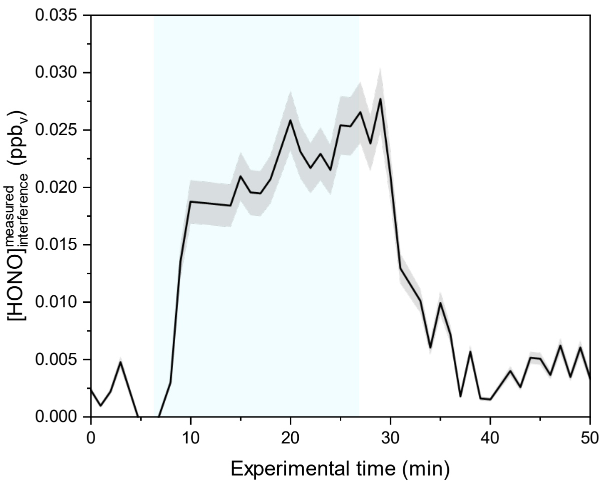

Figure S10: HONO concentration measured by the interference channel of the LOPAP during experiments with droplets containing 1 mM $NaNO_2$ at pH 10.5. This signal considered as HONO is solely attributed to an interferent and not a real HONO concentration. The spraying time is represented by the blue shaded region. In the LOPAP this channel should be constant if only HONO is present in the gas phase.

Table S5: Proposed mechanistic pathways for NO_2^- specific chemistry at the air-water interface.

	Mechanism	Reaction	Rate constant ($M^{-1}\ s^{-1}$)
NO_2^- Droplets	Formation of H_2O_2	$OH^- \rightleftharpoons \cdot OH + e^-$	-
		$\cdot OH + \cdot OH \rightarrow H_2O_2$	5.2×10^9 [6]
		$O_2 + e^- \rightarrow O_2^-$	1.9×10^{10} [7]
		$O_2^- + H^+ \rightleftharpoons HO_2^\cdot$	5.0×10^{10} [8]
		$HO_2^\cdot + HO_2^\cdot \rightarrow H_2O_2 + O_2$	8.3×10^5 [9]
		$H^+ + e^- \rightarrow H^\cdot$	2.4×10^{10} [10]
		$HO_2^\cdot + H^\cdot \rightarrow H_2O_2$	1.0×10^6 [11]
	NO_2^- Chemistry	$NO_2^- \rightleftharpoons NO_2^\cdot + e^-$	-
		$NO_2^- + \cdot OH \rightarrow NO_2^\cdot$	1.1×10^{10} [12]
		$NO_2^\cdot + \cdot OH \rightarrow HNO_3$	1.2×10^{10} [13]
		$HNO_3 \rightleftharpoons H^+ + NO_3^-$	
		$NO_2^- + e^- \rightarrow NO_2^{2-}$	3.5×10^9 [14,15]
		$NO_2^\cdot + O_2^- \rightarrow NO_2^-$	1.0×10^8 [16]
		$NO_2^{2-} + H_2O \rightarrow NO + 2\ OH^-$	5.5×10^4 [17]
		$NO_2^- + H^\cdot \rightarrow NO + OH^-$	7.1×10^8 [18]
		$NO_2^\cdot + O_2^- \rightarrow O_2NO_2^-$	4.5×10^9 [19]
		$O_2NO_2^- \rightarrow NO_2^- + O_2$	1.0 [19]
		$NO + O_2^- \rightarrow ONO_2^-$	4.3×10^9 [20]

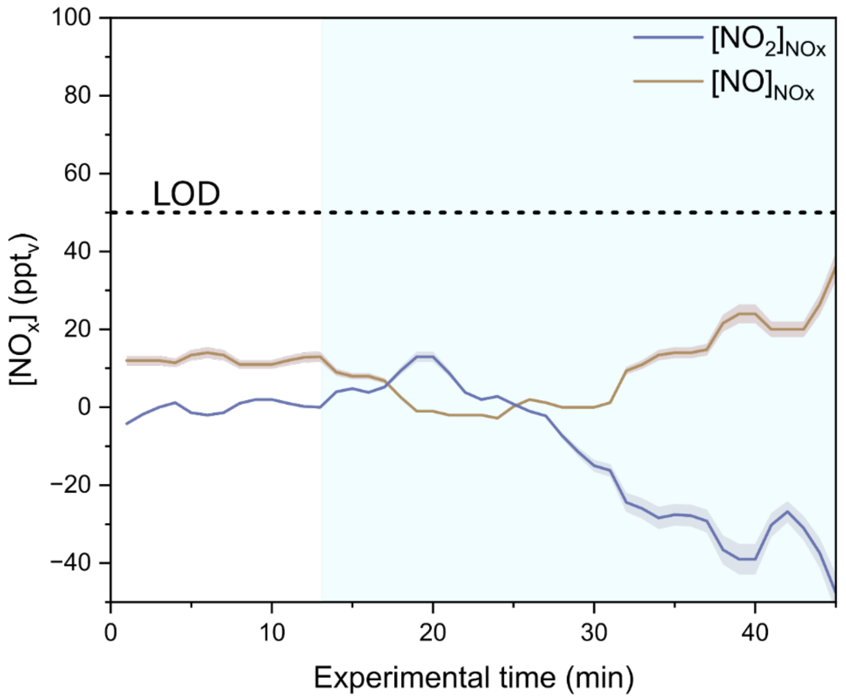

Figure S11: NO_x concentration measured by the NO_x analyzer during $NaNO_3$ droplets. The blue shaded region represents the spraying time. The dotted line represents the LOD of the NO_x analyzer

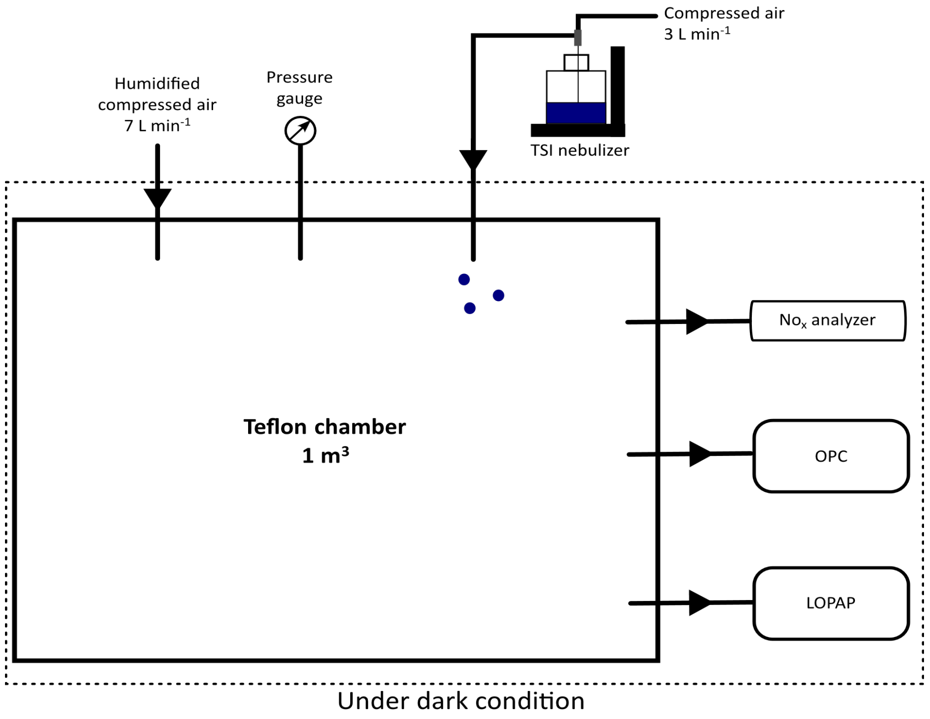

Figure S12: Atmospheric chamber experimental setup.

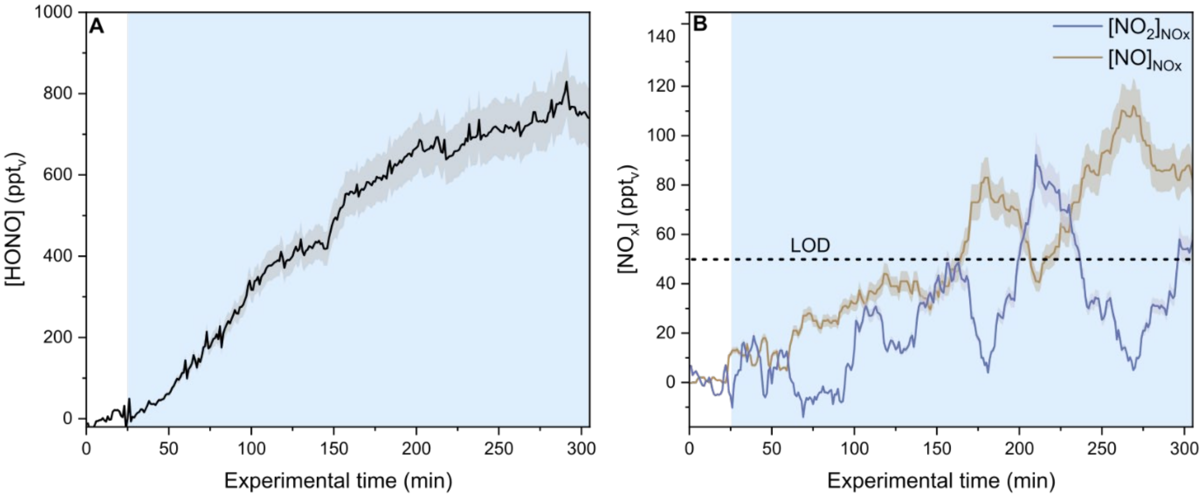

Figure S13: A) HONO measurement and B) NO_x measurement for droplets containing 500 mM NO_3^- in the atmospheric simulation chamber. The spraying time is represented by blue shaded region and the black dots line in B represent the limit of detection (LOD) of the NO_x analyzer.

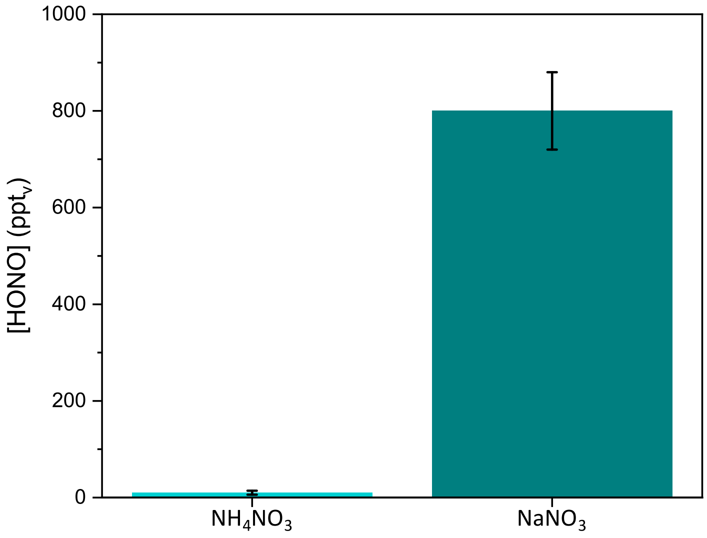

Figure S14: HONO concentration measured in the case of 500 mM $NaNO_3$ and NH_4NO_3 during experiments in the atmospheric simulation chamber (Figure S12).

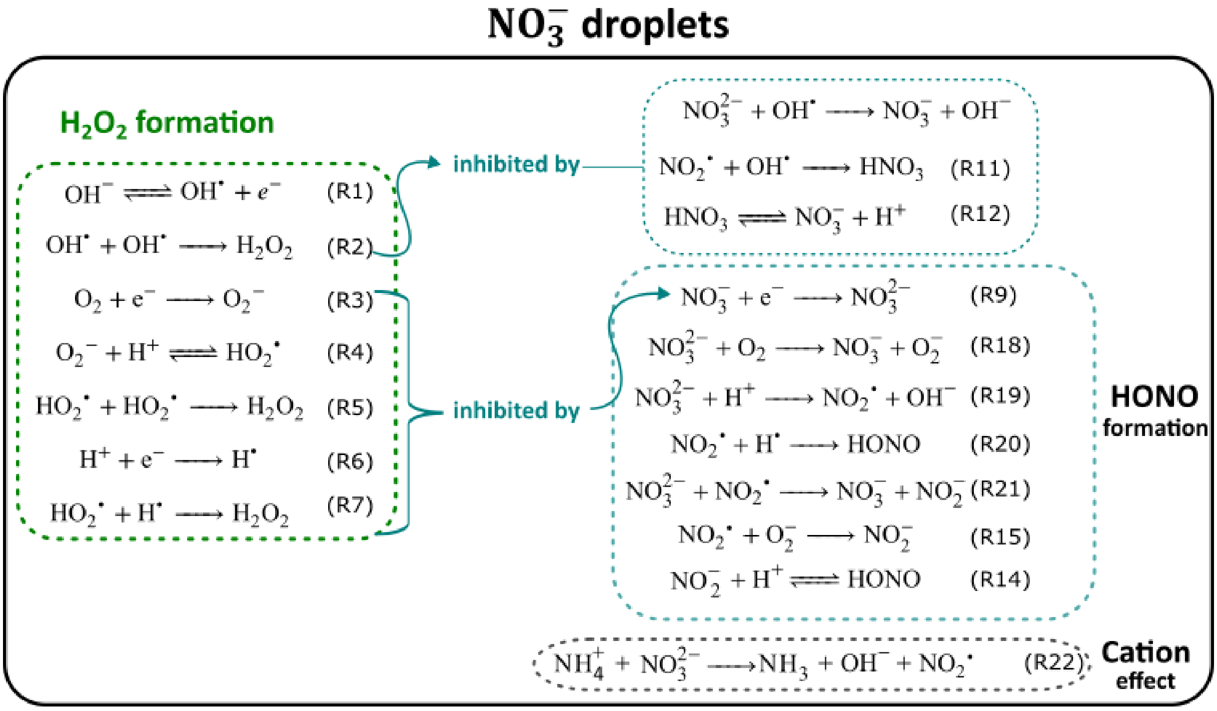

Figure S15: Proposed mechanism for NO_3^- spontaneous chemistry at the air-water interface under dark condition.

Table S6: Proposed mechanistic pathways for NO_3^- specific chemistry at the air-water interface

	Mechanism	Reaction	Rate constant (M^{-1} s^{-1})
NO_3^- Droplets	**Formation of H_2O_2**	$OH^- \rightleftharpoons {}^{\cdot}OH + e^-$	-
		${}^{\cdot}OH + {}^{\cdot}OH \rightarrow H_2O_2$	5.2×10^{9} [6]
		$O_2 + e^- \rightarrow O_2^-$	1.9×10^{10} [7]
		$O_2^- + H^+ \rightleftharpoons HO_2^{\cdot}$	5.0×10^{10} [8]
		$HO_2^{\cdot} + HO_2^{\cdot} \rightarrow H_2O_2 + O_2$	8.3×10^{5} [9]
		$H^+ + e^- \rightarrow H^{\cdot}$	2.4×10^{10} [10]
		$HO_2^{\cdot} + H^{\cdot} \rightarrow H_2O_2$	1.0×10^{6} [11]
	**NO_3^- Chemistry : HONO formation**	$NO_3^- + e^- \rightarrow NO_3^{2-}$	1.0×10^{10} [21,22]
		$NO_3^{2-} + O_2 \rightarrow NO_3^- + O_2^-$	2.0×10^{8} [23]
		$NO_3^{2-} + H^+ \rightarrow NO_2^{\cdot} + OH^-$	2.0×10^{10} [24]
		$NO_2^{\cdot} + H^{\cdot} \rightarrow HONO$	1.0×10^{10} [19]
		$NO_3^{2-} + NO_2^{\cdot} \rightarrow NO_3^- + NO_2^-$	-
		$NO_2^{\cdot} + O_2^- \rightarrow NO_2^-$	1.0×10^{8} [16]
		$NO_2^- + H^+ \rightleftharpoons HONO$	-
	**NO_3^- Chemistry : Side reaction**	$NO_3^{2-} + {}^{\cdot}OH \rightarrow NO_3^- + OH^-$	3.0×10^{9} [19]
		$NO_2^{\cdot} + {}^{\cdot}OH \rightarrow HNO_3$	1.2×10^{10} [13]
		$HNO_3 \rightleftharpoons H^+ + NO_3^-$	-
	**NO_3^- Chemistry : Cation effect**	$NH_4^+ + NO_3^{2-} \rightleftharpoons NH_3 + OH^- + NO_2$	2.0×10^{8} [24]

References

(1) Davies, C. W. 397. The Extent of Dissociation of Salts in Water. Part VIII. An Equation for the Mean Ionic Activity Coefficient of an Electrolyte in Water, and a Revision of the Dissociation Constants of Some Sulphates. *J. Chem. Soc.* **1938**, 2093. https://doi.org/10.1039/jr9380002093.

(2) Villena, G.; Kleffmann, J. A Source for the Continuous Generation of Pure And\hack\break Quantifiable HONO Mixtures. *Atmospheric Measurement Techniques* **2022**, *15* (3), 627–637. https://doi.org/10.5194/amt-15-627-2022.

(3) Riordan, E.; Minogue, N.; Healy, D.; O'Driscol, P.; Sodeau, J. R. Spectroscopic and Optimization Modeling Study of Nitrous Acid in Aqueous Solution. *J. Phys. Chem. A* **2005**, *109* (5), 779–786. https://doi.org/10.1021/jp040269v.

(4) Cohen, L.; Quant, M. I.; Donaldson, D. J. Real-Time Measurements of PH Changes in Single, Acoustically Levitated Droplets Due to Atmospheric Multiphase Chemistry. *ACS Earth Space Chem.* **2020**, *4* (6), 854–861. https://doi.org/10.1021/acsearthspacechem.0c00041.

(5) Angelaki, M.; d'Erceville, J.; Donaldson, D. J.; George, C. PH Affects the Spontaneous Formation of $H_2 O_2$ at the Air–Water Interfaces. *J. Am. Chem. Soc.* **2024**, *146* (38), 25889–25893. https://doi.org/10.1021/jacs.4c07356.

(6) Pagsberg, P.; Christensen, H.; Rabani, J.; Nilsson, G.; Fenger, J.; Nielsen, S. O. Far-Ultraviolet Spectra of Hydrogen and Hydroxyl Radicals from Pulse Radiolysis of Aqueous Solutions. Direct Measurement of the Rate of H + H. *J. Phys. Chem.* **1969**, *73* (4), 1029–1038. https://doi.org/10.1021/j100724a044.

(7) Elliot, A. J. A Pulse Radiolysis Study of the Temperature Dependence of Reactions Involving H, OH and e-Aq in Aqueous Solutions. *International Journal of Radiation Applications and Instrumentation. Part C. Radiation Physics and Chemistry* **1989**, *34* (5), 753–758. https://doi.org/10.1016/1359-0197(89)90279-8.

(8) Ilan, Y.; Rabani, J. On Some Fundamental Reactions in Radiation Chemistry: Nanosecond Pulse Radiolysis. *International Journal for Radiation Physics and Chemistry* **1976**, *8* (5), 609–611. https://doi.org/10.1016/0020-7055(76)90030-9.

(9) Bielski, B. H. J.; Cabelli, D. E.; Arudi, R. L.; Ross, A. B. Reactivity of HO2/O−2 Radicals in Aqueous Solution. *Journal of Physical and Chemical Reference Data* **1985**, *14* (4), 1041–1100. https://doi.org/10.1063/1.555739.

(10) Gordon, Sheffield.; Hart, E. J.; Matheson, M. S.; Rabani, Joseph.; Thomas, J. K. **Reaction Constants of the Hydrated Electron**. *J. Am. Chem. Soc.* **1963**, *85* (10), 1375–1377. https://doi.org/10.1021/ja00893a002.

(11) Thomas, J. K. THE RATE CONSTANTS FOR H ATOM REACTIONS IN AQUEOUS SOLUTIONS[1]. *J. Phys. Chem.* **1963**, *67* (12), 2593–2595. https://doi.org/10.1021/j100806a022.

(12) Treinin, A.; Hayon, E. Absorption Spectra and Reaction Kinetics of NO2, N2O3, and N2O4 in Aqueous Solution. *J. Am. Chem. Soc.* **1970**, *92* (20), 5821–5828. https://doi.org/10.1021/ja00723a001.

(13) Barat, F.; Gilles, L.; Hickel, B.; Sutton, J. Flash Photolysis of the Nitrate Ion in Aqueous Solution: Excitation at 200 Nm. *J. Chem. Soc., A* **1970**, 1982. https://doi.org/10.1039/j19700001982.

(14) Elliot, A. J.; McCracken, D. R.; Buxton, G. V.; Wood, N. D. Estimation of Rate Constants for Near-Diffusion-Controlled Reactions in Water at High Temperatures. *Faraday Trans.* **1990**, *86* (9), 1539. https://doi.org/10.1039/ft9908601539.

(15) Baxendale, J. H.; Fielden, E. M.; Keene, J. P. The Pulse Radiolysis of Aqueous Solutions of Some Inorganic Compounds. *Proceedings of the Royal Society of London. Series A. Mathematical and Physical Sciences* **1965**, *286* (1406), 320–336. https://doi.org/10.1098/rspa.1965.0147.

(16) Warneck, P.; Wurzinger, C. Product Quantum Yields for the 305-Nm Photodecomposition of Nitrate in Aqueous Solution. *J. Phys. Chem.* **1988**, *92* (22), 6278–6283. https://doi.org/10.1021/j100333a022.

(17) Grätzel, M.; Henglein, A.; Lilie, J.; Beck, G. Pulsradiolytische Untersuchung Einiger Elementarprozesse Der Oxydation Und Reduktion Des Nitritions. *Ber Bunsenges Phys Chem* **1969**, *73* (7), 646–653. https://doi.org/10.1002/bbpc.19690730707.
(18) Smaller, B.; Avery, E. C.; Remko, J. R. EPR Pulse Radiolysis Studies of the Hydrogen Atom in Aqueous Solution. I. Reactivity of the Hydrogen Atom. *The Journal of Chemical Physics* **1971**, *55* (5), 2414–2418. https://doi.org/10.1063/1.1676425.
(19) Loegager, T.; Sehested, K. Formation and Decay of Peroxynitrous Acid: A Pulse Radiolysis Study. *J. Phys. Chem.* **1993**, *97* (25), 6664–6669. https://doi.org/10.1021/j100127a016.
(20) Goldstein, S.; Czapski, G. The Reaction of NO· with O2·− and HO2·−: A Pulse Radiolysis Study. *Free Radical Biology and Medicine* **1995**, *19* (4), 505–510. https://doi.org/10.1016/0891-5849(95)00034-U.
(21) Hentz, R. R.; Farhataziz; Hansen, E. M. Pulse Radiolysis of Liquids at High Pressures. II. Diffusion-Controlled Reactions of the Hydrated Electron. *The Journal of Chemical Physics* **1972**, *56* (9), 4485–4488. https://doi.org/10.1063/1.1677893.
(22) Peled, E.; Czapski, G. Molecular Hydrogen Formation (GH2) in the Radiation Chemistry of Aqueous Solutions. *J. Phys. Chem.* **1970**, *74* (15), 2903–2911. https://doi.org/10.1021/j100709a010.
(23) Forni, L. G.; Mora-Arellano, V. O.; Packer, J. E.; Willson, R. L. Nitrogen Dioxide and Related Free Radicals: Electron-Transfer Reactions with Organic Compounds in Solutions Containing Nitrite or Nitrate. *J. Chem. Soc., Perkin Trans. 2* **1986**, No. 1, 1. https://doi.org/10.1039/p29860000001.
(24) Benderskii, V. A.; Krivenko, A. G.; Ponomarev, E. A.; Fedorovich, N. V. Rate Constants of Protonation of the Ion Radical NO32−. *Elektrokhimiya* **1987**, *23*, 1435–1439.
